# A Motivational Interviewing Chatbot With Generative Reflections for Increasing Readiness to Quit Smoking: Iterative Development Study

**DOI:** 10.2196/49132

**Published:** 2023-10-17

**Authors:** Andrew Brown, Ash Tanuj Kumar, Osnat Melamed, Imtihan Ahmed, Yu Hao Wang, Arnaud Deza, Marc Morcos, Leon Zhu, Marta Maslej, Nadia Minian, Vidya Sujaya, Jodi Wolff, Olivia Doggett, Mathew Iantorno, Matt Ratto, Peter Selby, Jonathan Rose

**Affiliations:** 1 The Edward S Rogers Sr Department of Electrical & Computer Engineering University of Toronto Toronto, ON Canada; 2 INTREPID Lab Centre for Addiction and Mental Health Toronto, ON Canada; 3 Department of Family and Community Medicine University of Toronto Toronto, ON Canada; 4 Krembil Centre for Neuroinformatics Centre for Addiction and Mental Health Toronto, ON Canada; 5 Campbell Family Mental Health Research Institute Centre for Addiction and Mental Health Toronto, ON Canada; 6 Department of Pharmacology and Toxicology University of Toronto Toronto, ON Canada; 7 Institute of Medical Sciences University of Toronto Toronto, ON Canada; 8 Faculty of Information University of Toronto Toronto, ON Canada; 9 Dalla Lana School of Public Health University of Toronto Toronto, ON Canada

**Keywords:** conversational agents, chatbots, behavior change, smoking cessation, motivational interviewing, deep learning, natural language processing, transformers, generative artificial intelligence, artificial intelligence, AI

## Abstract

**Background:**

The motivational interviewing (MI) approach has been shown to help move ambivalent smokers toward the decision to quit smoking. There have been several attempts to broaden access to MI through text-based chatbots. These typically use scripted responses to client statements, but such nonspecific responses have been shown to reduce effectiveness. Recent advances in natural language processing provide a new way to create responses that are specific to a client’s statements, using a *generative* language model.

**Objective:**

This study aimed to design, evolve, and measure the effectiveness of a chatbot system that can guide ambivalent people who smoke toward the decision to quit smoking with MI-style generative reflections.

**Methods:**

Over time, 4 different MI chatbot versions were evolved, and each version was tested with a separate group of ambivalent smokers. A total of 349 smokers were recruited through a web-based recruitment platform. The first chatbot version only asked questions without reflections on the answers. The second version asked the questions and provided reflections with an initial version of the reflection generator. The third version used an improved reflection generator, and the fourth version added extended interaction on some of the questions. Participants’ readiness to quit was measured before the conversation and 1 week later using an 11-point scale that measured 3 attributes related to smoking cessation: readiness, confidence, and importance. The number of quit attempts made in the week before the conversation and the week after was surveyed; in addition, participants rated the perceived empathy of the chatbot. The main body of the conversation consists of 5 scripted questions, responses from participants, and (for 3 of the 4 versions) generated reflections. A pretrained transformer-based neural network was fine-tuned on examples of high-quality reflections to generate MI reflections.

**Results:**

The increase in average confidence using the nongenerative version was 1.0 (SD 2.0; *P*=.001), whereas for the 3 generative versions, the increases ranged from 1.2 to 1.3 (SD 2.0-2.3; *P*<.001). The extended conversation with improved generative reflections was the only version associated with a significant increase in average importance (0.7, SD 2.0; *P*<.001) and readiness (0.4, SD 1.7; *P*=.01). The enhanced reflection and extended conversations exhibited significantly better perceived empathy than the nongenerative conversation (*P*=.02 and *P*=.004, respectively). The number of quit attempts did not significantly change between the week before the conversation and the week after across all 4 conversations.

**Conclusions:**

The results suggest that generative reflections increase the impact of a conversation on readiness to quit smoking 1 week later, although a significant portion of the impact seen so far can be achieved by only asking questions without the reflections. These results support further evolution of the chatbot conversation and can serve as a basis for comparison against more advanced versions.

## Introduction

### Background

Tobacco use is the leading preventable cause of premature death in Canada, killing 45,000 Canadians every year [1], with 4.6 million Canadians ensnared by the addiction [2]. A key step in smoking cessation is the decision by the smoker to quit; yet, 80% of all smokers are *ambivalent* [3] about quitting and make no current effort to stop smoking [4]. In this paper, we report on an automated method to engage ambivalent smokers in a web-based conversation with the aim of moving them toward the decision to quit. This goal is distinct from smoking cessation efforts that assume the smoker is ready and willing to quit; however, the decision to quit is a necessary precursor of any quit attempt.

Smokers can be guided toward the decision to quit by a widely used talk therapy approach known as motivational interviewing (MI) [5]. MI guides individuals toward healthy behavior change by helping them explore their ambivalence. As MI relies on highly trained clinicians working at hospitals and specialized clinics, it is both expensive and difficult to access. Clinicians are usually engaged only *after* a health issue occurs, whereas earlier engagement with a more accessible chatbot could improve health outcomes and even prevent illness or death. For every 2 smokers helped to quit, 1 life is saved from a tobacco related death [6]. If it were possible to *automate* an MI-style conversation and deploy it directly to smokers on the web, smokers could be helped much sooner.

However, it is challenging for a machine to achieve the level of understanding and facility needed to practice MI. Prior efforts at automated therapy, beginning with ELIZA [7] and proceeding through many generations of dialogue systems [8-10], suffer from 2 key issues. First, because most of the outgoing text is scripted, these systems have difficulty responding to the specific things that an individual says. These responses are often seen by users as either repetitive or too generic [11]. Second, many chatbots do not permit free-form text input, which prevents users from expressing themselves fully. Recent dramatic advances in *natural language generation* [6,12-14] have produced language models that can generate very humanlike responses that are more relevant to the free-form dialogue of a human.

In this paper, we present the design of several versions of a chatbot, called *MIBot*, that makes use of these new kinds of language models to generate context-specific responses to user statements in combination with scripted interactions. We also present a scientific infrastructure for measuring the impact of MIBot on recruited smokers.

This project is a collaboration among a group of MI researchers and clinicians, computer engineers, and social scientists. The clinicians, who work with patients to resolve their ambivalence toward quitting smoking using the MI approach, bring their expertise to the initial design and iterative steps of the chatbot. The computer engineers bring experience in methods of software and natural language generation. The social scientists draw on perspectives in human-computer interaction and notions of trust. The ongoing decision-making and iterative improvement of the chatbot described in this paper follows the principles and rationale of a co-design process [15]. As noted by Donia and Shaw [15], such processes are key to appropriate outcomes when dealing with health care and artificial intelligence technologies. The group has met biweekly for >2 years, and each subgroup has sought to learn from the other so that a true cross-disciplinary interaction and purposeful evolution of the chatbot take place. The interactions explore the tension between what is needed in a successful MI conversation and what is possible to automate in limited time. We have all enjoyed learning across traditional disciplinary boundaries.

This paper is organized as follows: the *Prior Related Work* subsection reviews MI and related work on conversational agents and chatbots, as well as the relevant new capabilities in natural language generation. The *Methods* section describes the 4 versions of the chatbot and the training of the neural networks needed to provide the generative responses, as well as the recruitment and measurement methods. The *Results* section presents the results from the 4 versions of the chatbot, and the *Discussion* section contrasts these results and the impact of the different versions.

### Prior Related Work

#### The MI Approach

MI is a counseling approach that helps patients increase motivation toward changing unhealthy behaviors, including addictions [5]. The goal of MI is to move a person away from ambivalence, a conflicted state where opposing attitudes or feelings coexist in an individual toward changing a behavior. MI counselors use a structured conversation that includes open-ended questions and reflective listening, which encourage a patient to contemplate the roots of a behavior and guide them toward overcoming their ambivalence.

A core skill used by MI practitioners is reflective listening [5,16,17]. A reflective listener responds to patient statements with words that both reflect what is said and guide the patient toward continued exploration of their thoughts and feelings about change. These responses, called *reflections* in MI, can be simple or complex. A simple reflection repeats or rephrases what the patient has said to convey understanding and invite continuation of the conversation. A complex reflection attempts to infer something relevant to the prior conversation or guess something that might be relevant to a recent utterance, which also invites continued contemplation on the part of the patient.

MI has been shown to be a successful therapeutic tool for motivating many behavior changes [18], including smoking cessation [19,20].

#### Natural Language Processing and Generative Networks

Natural language processing (NLP) is a subfield of linguistics, computer science, and machine learning concerned with the interactions between computers and human (natural) languages [21]. Over the last 11 years, there have been significant advances in the field of NLP [8]. A key step was the invention of limited-sized word vectors or embeddings, in which it has been shown that a small-sized vector of real numbers (from 50 to 300) could represent the meaning of individual words or parts of words (called *tokens*) [22,23]. Hereinafter, for simplicity, we will refer to these elements as words. These word vectors make it possible to know whether 2 words have similar meaning through a numerical comparison and have led to significant advances in the subfields of speech recognition, natural language understanding (NLU), question answering (QA), and natural language generation [13,14,21,24,25].

In particular, the advent of the *transformer*-based neural network has dramatically improved the state of the art in most NLP subfields [12,13]. This success comes when these networks are scaled up to relatively large sizes and *trained* on large amounts of human-written text; for example, OpenAI’s generative pretrained transformer 2 (GPT-2) model consists of 1.5 billion parameters that are trained on 40 GB of text gathered from various domains [13].

Transformer networks can be used to both analyze and *generate* language given an input text. It is this generative capability that we make extensive use of in this work. This was a relatively unknown capability that recently came to broad impact with the release of ChatGPT [26] in November 2022.

There now exist many such large language models that have been fully *pretrained* on massive corpora of text gathered from several sources on the internet and elsewhere [13,14,27]. A common use case in the field of deep learning and NLP is to take such pretrained models and *fine-tune* [8] them for a specific prediction task that takes language as input. To *fine-tune* a model means to train it on a (typically much smaller) data set to become more proficient at a specific task.

#### MI Chatbots

There have been several prior attempts to automate MI using a chatbot across different domains, including stress management, sex health education, and smoking cessation [19,20,28-32]; for example, Park et al [28] designed a conversational sequence using MI to aid in stress management. This conversation was deployed to 30 graduate students to compare its efficacy with that of human-to-human MI. It posed thought-provoking questions combined with scripted reflections. Participants reported that they were satisfied with the evocative questions but were dissatisfied with the prewritten reflective statements.

Almusharraf et al [29] and Almusharraf [30], the predecessors of this work, designed and tested an MI chatbot used for motivating smoking cessation. The chatbot used NLP classifiers to select scripted responses that guided a client through the conversation. Both the questions and the reflections in this chatbot were scripted. In a study involving 97 participants, Almusharraf et al [29] found that the average confidence to quit on an 11-point scale increased 1 week after the conversation by 0.8 (*P*<.001).

He et al [19] created both an MI chatbot and a non-MI chatbot to investigate whether chatbots can motivate smoking cessation. In an experiment with 153 participants, differences in motivation to quit smoking and perceived empathy were compared between a chatbot that used MI and one that did not. Both chatbots used evoking questions and prewritten statements for reflections. There were no significant differences between the chatbots on engagement, therapeutic alliance, or perceived empathy. Notably, for both chatbots, participants reported significantly increased motivation to quit smoking.

The chatbots developed to date used scripted statements based on keyword detection or neural net–based classification of the users’ utterances. Scripted responses are often interpreted by humans as generic and repetitive [11], making it possible that the scripted reflections used in prior work contributed to poor user satisfaction and perceived empathy. We hypothesize that a chatbot capable of delivering context-specific MI reflections will better motivate smokers to move toward the decision to quit.

Some works exploring the use of generated reflections within MI-style conversational agents demonstrate this capacity; for example, Shen et al [33] showed how transformer-based models can produce context-specific generative reflections. Although these reflections were used to train practitioners and were not patient facing, they highlight the capacity of transformer-based models to produce good-quality patient-specific reflections. Similarly, Saiyed et al [20] created a technology-assisted MI chatbot for smoking cessation. The chatbot was designed to onboard participants, use MI, and refer participants to human-to-human treatment. It used intent classifiers and transformers to understand and generate utterances, including MI reflections. In a pilot trial of 34 smokers, participants reported that the chatbot had a strong competency in MI but only scored 3 out of 5 on user satisfaction, leaving room for improvement.

The goal of this study was to determine the impact of several versions of an MI-oriented chatbot, which uses generative reflections, on moving smokers toward the decision to quit smoking. Most of the prior work used scripted statements based on keyword detection or neural net–based classification of the users’ utterances. It is possible that scripted reflections caused prior work to score poorly on user satisfaction and perceived empathy. We hypothesize that context-specific MI reflections, such as those developed by Shen et al [33], deployed in a chatbot intervention will better motivate smokers to move toward the decision to quit.

## Methods

In this section, we describe the overall structure of the MIBot conversation and its several versions and provide the specifics of how the generative model is trained to provide MI-style reflections. We also describe how the effectiveness of the conversation is measured.

### Overall Chatbot System Study Design

**Figure 1:**
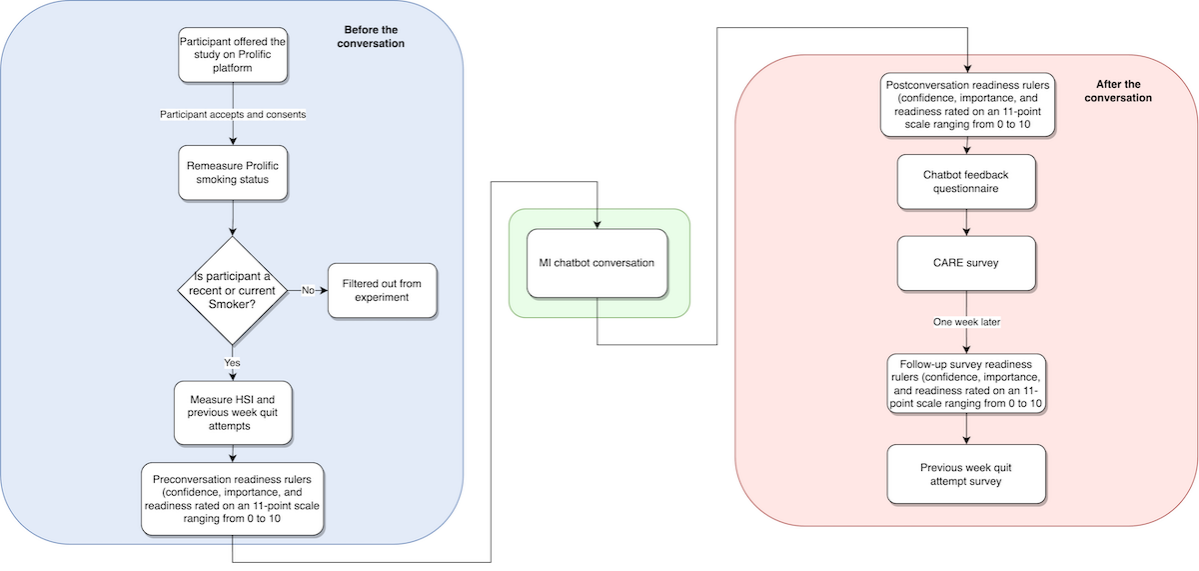
Overall design of each study for each chatbot version. CARE: consultation and relational empathy; HSI: Heaviness of Smoking Index; MI: motivational interviewing.

Figure 1 illustrates the procedure used to evaluate each version of the chatbot. Details of the steps are described in Textbox 1.

The following subsections provide the details of the interaction and evaluation process.

**Details of the steps involved in the evaluation of the chatbot.**

- Participants are recruited through the web-based Prolific [34] paid-recruitment system, the details of which are provided in *Participant Recruitment and Screening* subsection. Each participant who is offered the opportunity to participate (by Prolific) is asked to review an informed consent document. The interaction proceeds if the participant provides consent.
- Participants are taken to a custom website and asked several questions, after which they fill out 3 surveys on readiness to quit, heaviness of smoking, and number of quit attempts, as described in the *Preconversation Surveys and Screening* subsection.
- Next, the participant is presented with a text chat window in which the chatbot, called *MIBot*, begins to interact with them. The conversation begins with an introductory section, after which the participant is asked whether they wish to chat about smoking, as part of a motivational interviewing–style permission-asking approach [5]. If the participant agrees, the conversation continues; otherwise, it terminates.
- The core conversation, described in the *Core Conversation* subsection, ensues.
- After the conversation, the participant is asked to respond to another readiness-to-quit survey, the consultation and relational empathy measure, and other qualitative questions, as described in the *Postconversation Survey* subsection.
- Finally, the participant is directed back to the Prolific system so that it can record the successful conclusion of the tasks.
- One week *after* the end of the conversation, the participant is invited to a second task (also within the Prolific system), which is to answer the survey and questions described in the *One-Week Follow-Up Survey* subsection. Participants are not paid unless they fill out this follow-up survey, and their submission is reviewed for data quality.

### Core Conversation

The core conversation consists of 5 open-ended questions that make use of the “running head start” [5] method of MI. The underlying theory of the running head start method is that ambivalent smokers spend very little time thinking about their smoking addiction and do it by habit [35]. Therefore, a key first step is to bring the habit to their attention and ask them to contemplate it. Here, a clinician inquires what participants like about smoking and what they do not like and then uses these reasons as a basis for further discussion and contemplation. This approach is realized through asking these five questions:

1. To start, what is the thing you like most about smoking?
2. Now, what is the thing you like least about smoking?
3. Now, what is one thing about your smoking habit that you would like to change?
4. What will it look like when you have made this change in your smoking habit?
5. Finally, what are the steps you need to take to make this change?

The first 2 questions are based on the running head start approach, and the subsequent 3 questions attempt to stimulate contemplation around the addiction.

One important aspect of this chatbot is that participants respond with *free-form text*. This means that they can provide any textual response in English as opposed to making a selection from scripted responses [10]. In allowing free-form responses, the conversation may more closely align with a human–clinical provider conversation.

In this paper, we will describe and evaluate several versions of the conversation. For most of the versions, the chatbot generates an MI-style *reflection* of the free-form response to each question that is specific to the words of the response, as described in the *Prior Related Work* subsection. Here, we use a fine-tuned transformer-based neural network [13] to generate that reflection. The data and training of this neural network are described in the *Reflection Generation Design and Training* subsection.

After each reflection is provided, the chatbot (in most versions) asks, “Did that make sense?” If the participant responds in a way that indicates *yes*, the chatbot offers a thank you. If the participant’s answer is equivalent to a *no*, the chatbot thanks the participant for helping it improve. As the participant can write a free-form text answer, they may respond to the chatbot’s question in many ways (eg, offering a longer, possibly corrective answer).

### Chatbot Versions

The ongoing goal of the long-term project is to continuously improve the chatbot system through iteration, and, in this paper, we report on the impact that 4 different versions have on readiness to quit smoking in recruited participants. It should be noted that this means that participants are not randomized to different conditions, and the results will be subject to cohort effects. In future work, once we believe that the chatbot is having a sufficient impact, we will measure it against a control. Each new version increases the complexity of the interaction. Table 1 provides a short description of each version. The differences among the versions are more readily seen in Multimedia Appendix 1, which provides an example of the full conversation for each version, taken from actual conversations with participants.

**Table 1:** Chatbot versions.

Version	Description	Period of experiment
MIBot v4.7	Asks *just* the 5 questions shown in the *Core Conversation* subsection but *does not* provide reflections (instead, responds “Thank you”)	July 26, 2022, to August 2, 2022
MIBot v5.0	Asks the 5 questions and provides MI^a^-style reflective answers, as described in the *Core Conversation* subsection; uses the early version of the generator, described in the *Reflection Generation and Training* subsection	August 12 to 19, 2022
MIBot v5.1	Same as v5.0 but uses the significantly improved reflection generator, described in the *Reflection Generation and Training* subsection	August 16 to 23, 2022
MIBot v5.2	On the basis of v5.1, with extensions to the sequence of questions 1 and 2; in addition, if the answer to question 3 relates to *reduction* of smoking, it changes question 4 to be specifically about reduction; furthermore, it extends the interaction around question 3, as described in the *Enhanced Conversation* subsection	November 22 to 29, 2022

^a^MI: motivational interviewing.

### Enhanced Conversation Structure

We have described the 4 versions of the chatbot in Table 1, but MIBot v5.2 needs to be presented in more detail because of its increased complexity. The structure of the enhanced conversation is presented in Textbox 2.

**Structure of the enhanced conversation (MIBot v5.2).**

- After the reflection is generated for question 1 and question 2, the chatbot asks *what else* the participant likes (or dislikes in the case of question 2) about smoking. If the answer provides more reasons, these are also reflected. If there are no further reasons, then the chatbot moves on to the next question without generating a reflection or asking for validation of the previous reflections.
- If the participant’s response to question 3 is related to the *reduction* of smoking, a common answer, then the chatbot switches to a different dialogue stream. The following question arises next: “It’s great to hear you want to reduce your smoking. What would it look like when you have reduced your smoking addiction?” After the reflection of the response to this question, the chatbot waits silently for 30 s to encourage the participant to respond on their own. If participants do not respond within 30 s, the chatbot prompts a response by asking the following question: “Could you elaborate on what I said?”

### Preconversation Surveys and Screening

Before interacting with the chatbot, to confirm the participant’s smoking status and ensure that this status has not changed since the administration of Prolific’s own screening survey, each participant is first asked to respond (again) to the same screening question administered by Prolific. Participants who provide a response that is inconsistent with that in their prior Prolific-administered survey, indicating that they do not identify as smokers, are not allowed to proceed with the study.

Next, the participant is asked to respond to a Heaviness of Smoking Index (HSI) survey [36], a validated survey that combines *cigarettes per day* (CPD) and *time to the first cigarette of the day* (TTF). After this, the participant indicates how many quit attempts they had made the previous week, as shown in Figure S1 in Multimedia Appendix 2.

Finally, participants are asked to fill out the readiness ruler [37] survey, a validated tool for tracking progress in MI sessions. Our version is shown in Figure S2 in Multimedia Appendix 2, which rates on an 11-point scale participants’ confidence that they could quit smoking now (ie, confidence), their readiness to quit now (ie, readiness), and how important they feel it is for them to quit (ie, importance).

Our protocol imposes a second screen that is based on the readiness ruler responses, as follows: since an MI conversation is targeted toward ambivalent smokers, it is not useful if the participant is already very confident that they will be able to quit; therefore, participants are only included if they have a confidence level of ≤5, with 1 exception: if those with a confidence level of >5 also rate the importance >5 points below this confidence level, there is a contradiction that implies the presence of ambivalence. These participants have confidence that they could quit, but they do not think it is important to do so and hence the contradiction.

### Postconversation Survey

After the conversation, the participant is asked to fill out a readiness ruler survey [37] similar to the one they completed before the conversation and then respond to the consultation and relational empathy (CARE) survey [38]. The latter is a validated tool developed to assess empathy in a primary care patient–provider relationship. Empathy in the therapeutic encounter is linked with patient satisfaction and positive health outcomes [39]. The CARE survey examines empathy in the encounter by asking patients to rate the ability of the provider (in this case, the chatbot) to (1) appreciate their perspective; (2) communicate back this understanding; and (3) given this understanding, be helpful to them. The CARE measure has 10 statements that are rated using a 6-point Likert scale with total scores ranging from 0 to 50.

Finally, the participants are asked to respond to the following qualitative questions:

1. What are 3 words that you would use to describe the chatbot?
2. What would you change about the conversation?
3. Did the conversation help you realize anything about your smoking behavior? Why or why not?

### One-Week Follow-Up Survey

One week after engaging in the conversation, participants are contacted through the Prolific platform to complete 2 more surveys: the first is a reprise of the readiness ruler survey [37] to determine to determine the effect on the principle outcome after a week has passed, which would be a more permanent effect than immediately after the conversation, and the second concerns 3 questions relating to quit attempts made during the preceding week. The first 2 questions are the same as the last 2 questions (Q3 and Q4) shown in Figure S1 in Multimedia Appendix 2, and the third is given in Figure S3 in Multimedia Appendix 2.

### Determining Whether Ambivalence Moved Toward Quitting or Continuing to Smoke

An underlying goal of this work is to help smokers begin to resolve their ambivalence toward smoking. It is possible to classify the data from each participant as belonging to 1 of 3 outcome categories: the participant moved toward changing their smoking addiction (toward quit class), they moved toward maintaining their smoking addiction (toward smoke class), or there was no change (same class).

To place participants into 1 of these 3 classes, we made use of 2 outcome data points. First, we computed the preconversation data to 1-week–later change in confidence from the readiness ruler, which can range in value from −6 to +10. A more positive value suggests a stronger move toward the quit class, whereas a negative value suggests a move toward the smoke class. Second, to gain a more accurate signal, we combined this number with a subjective evaluation of the participant’s answer to the third 1-week–later question: “Did the conversation help you realize anything about your smoking behavior? Why or why not?” If the participant stated that they realized something that helped them change their smoking addiction toward quitting and had a positive change in confidence, they were placed in the *toward quit* class. If the participant stated that they realized that they wished to sustain their smoking addiction, and the confidence change was negative, they were placed in the *toward smoke* class. If neither category fit, we placed them in the *same* class.

### Reflection Generation Training

One of the key contributions of this work is the novel way that MI-consistent reflections are generated in response to participant responses to the 5 questions shown in the *Core Conversation* subsection. Here, we made use of recent advances in NLP, and specifically in text generation, as described in the *Prior Related Work* subsection. The reflection generation neural network evolved from the one described in the studies by Ahmed [40] and Ahmed et al [41]. It makes use of the pretrained GPT-2 XL transformer-based neural network model [13], which is *fine-tuned*, as described in the *Prior Related Work* subsection. In this subsection, we provide more detail on how the generators used were trained.

There are 2 versions of the reflection generator that are evaluated in this study. The generators are fine-tuned using example sequences of text, which we call a triplet, consisting of a question, a response, and a reflection.

In version 1 of the generator, the fine-tuning question-and-response data set came from 2 sources: the first was our prior work [40,41], and the second data source was from earlier deployments of MIBot, before the creation of MIBot v4.7. The reflections used came from a variety of sources: from previous versions of this chatbot that were deemed to be acceptable MI reflections by MI-literate researchers or actual reflections produced by MI-literate researchers or MI-expert clinicians.

We used the *hit rate* metric to evaluate the quality of a generator, which is the number of MI-consistent reflections generated divided by the total number of reflections generated. The hit rate was measured on a validation set of question prompts and human responses that did not overlap with the training set. The MI-consistency of the reflection was judged by a single human rater trained in MI literacy. The hit rate of version 1 of the generator was approximately 76% (25/33) on a validation set of 33 prompts and responses. It is important to note that a hit rate of <100% means that some fraction of the generated responses will not be consistent with MI and may indeed make counterintuitive or simply wrong statements. In our experience, the most common type of error is a misstatement of the clear intent of the human; for example, when a user suggested that they would like to quit smoking, the chatbot would sometimes generate a reflection that implied that the user would like to continue smoking.

To address the rate of poor reflections, we developed version 2 of the generator with 2 significant enhancements. First, a larger set of 301 fine-tuning triplets were collected over approximately 10 months of deploying the chatbot, making use of the various responses from smokers who had been recruited in a similar manner, as described in the *Participant Recruitment and Screenin*g subsection. This second data set did not include any of the data from the earlier chatbot version [40,41]. Only MI-consistent reflections were used, which were sourced from MI clinicians, MI-literate researchers, or version 1 of the generator. The labeling and selection of the MI-consistent reflections were improved by using multiple human raters and a carefully controlled decision tree to determine the validity of the reflections. The new rating scheme itself was stricter than the one used in version 1, which caused the hit rate to go down—not because the generation was worse but because of the stricter rating. The hit rate of the new generator was measured to be 55.1% (166/301) on a set of reflections.

The second enhancement was the implementation of a separate *classifier* neural network, trained to determine whether a reflection is MI-consistent, given the prompt, response, and reflection in a triplet. The classifier was used to filter out poor reflections and therefore increase the overall hit rate of generation. This makes use of the fact that all modern neural network–style generators can easily generate many reflections because the generation process is carried out through *sampling* from a probability distribution [8]. The classifier is called the reflection quality classifier, and an earlier version of it is described in the studies by Ahmed [40] and Ahmed et al [41]. This reflection quality classifier, based on the Bidirectional Encoder Representations from Transformers (BERT) [27] pretrained neural network, was fine-tuned using a data set of 740 examples, both positive and negative. Using our validation data, we achieved an accuracy of 70.3% (71/101) on the question, prompt, and response triplets.

### Software System

**Figure 2:**
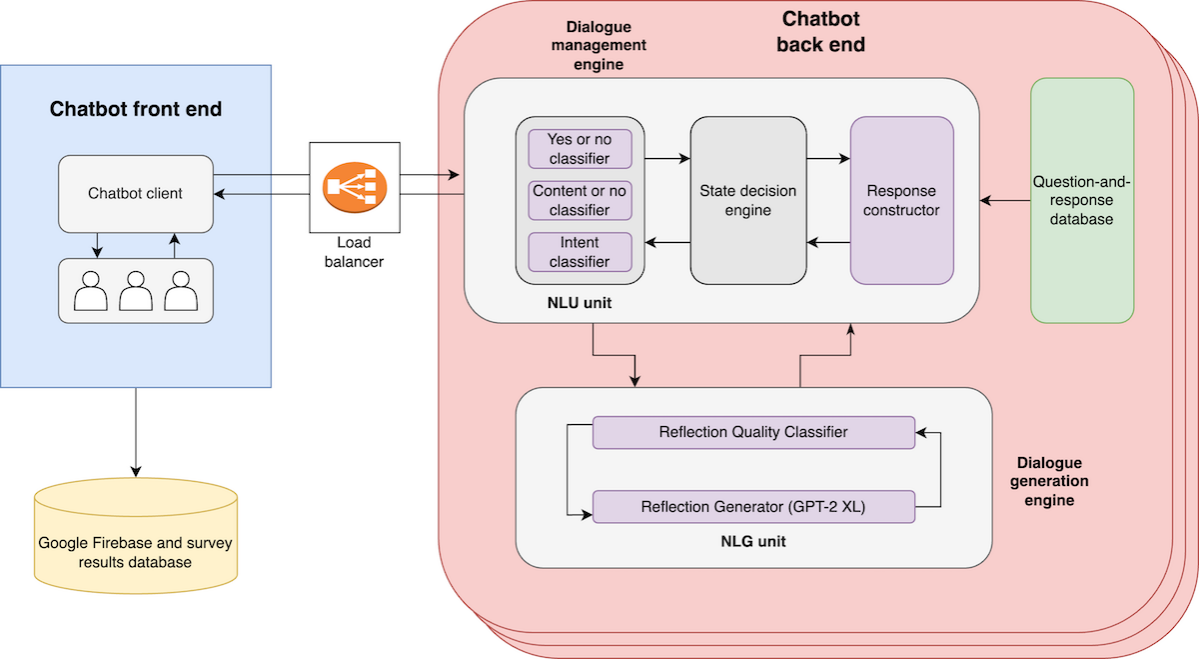
Chatbot architecture. GPT-2 XL: generative pretrained transformer 2 extra large; NLG: natural language generation; NLU: natural language understanding.

Figure 2 illustrates the structure of the software system used in the studies. Once the Prolific system transfers a participant to our system, they are brought to our chatbot front end, which exists on a web page. This web page connects to a backend database (based on Google Firebase [42]) that records the entire conversation and all data associated with the surveys and information that the participant provides. It also connects to the chatbot backend that contains compute accelerators capable of running the GPT-2 XL neural network in an amount of time that is acceptable to participants (provided by Amazon Elastic Compute Cloud [EC2] [43]). The chatbot backend is split into a dialogue management engine and a dialogue generation engine. The dialogue management engine uses an intent classifier, a yes or no classifier, and a content or no content classifier to classify incoming user utterances using NLU techniques and controls the current state and direction of the conversation. Responses are constructed using a combination of the dialogue generation engine and question-and-response database. The dialogue generation engine uses the GPT-2 XL neural network to generate MI reflections as described in the *Reflection Generation Training* subsection.

### Participant Recruitment and Screening

Participants were recruited through the Prolific [34] web-based recruitment system and were paid a total of £5 (US $6.25) for completing 2 tasks 1 week apart. The inclusion criteria, based on Prolific’s screening filters, were as follows: can be located in any country, minimum age 18 years, fluent in English, smoking status as per 1 of these 2 choices: “I am a current smoker (smoke at least 5 cigarettes a day and have smoked this amount for at least 1 year)” or “I am a recent smoker (smoke at least 5 cigarettes a day and have smoked this amount for <1 year),” and minimum approval rate of 90% on participant’s prior Prolific studies.

In addition, the Prolific system was set to recruit an equal number of men and women. However, because there was a subsequent screening of participants as described in the *Preconversation Surveys and Screening* subsection, the final number of participants was not balanced between men and women.

Data from the participants who completed part 1 and part 2 of the study were manually reviewed for data inclusion on the following criteria:

1. Participant properly filled out each survey metric with realistic values.
2. Participant responded to the chatbot with apparent honesty (eg, no toxic language or apparent ulterior motives).
3. Participant met the additional screening criteria as described in the *Preconversation Surveys and Screening* subsection.

### Ethics Approval

This research was approved by the University of Toronto Research Ethics Board under protocol number 35567, as amended, on June 29, 2022. All participants provided consent before participating in the study.

### Statistical Analysis

Significance testing was completed within and across chatbot versions. Within each chatbot version, we compared readiness ruler responses, quit attempts, and ambivalence resolution counts before and after the conversation. For readiness rulers, a 2-tailed *t* test was applied to examine changes in readiness ruler attributes (ie, readiness, confidence, and importance) from before the conversation to 1 week later. A Fisher exact test was used to evaluate significant changes in quit attempts from before the conversation to 1 week later. We also compared changes in readiness ruler attributes, CARE survey data, and reduction of smoking across chatbot versions. To compare readiness rulers and CARE survey data, a Welch *t* test was used, and to compare changes in reduction of smoking, we used a 2-sample proportion test (*z* test).

For all tests, a significance level of *P*<.05 was considered statistically significant. Statistical analysis was completed using the SciPy library for the Python programming language [44].

## Results

### Overview

This section reports on the impact of the 4 versions of the chatbot on recruited participants. We begin with the recruitment yield and data inclusion and then provide the demographics of participants. Next, we present the readiness rulers, quit attempts, CARE survey and HSI results, and ambivalence resolution counts.

### Recruitment Results

**Figure 3:**
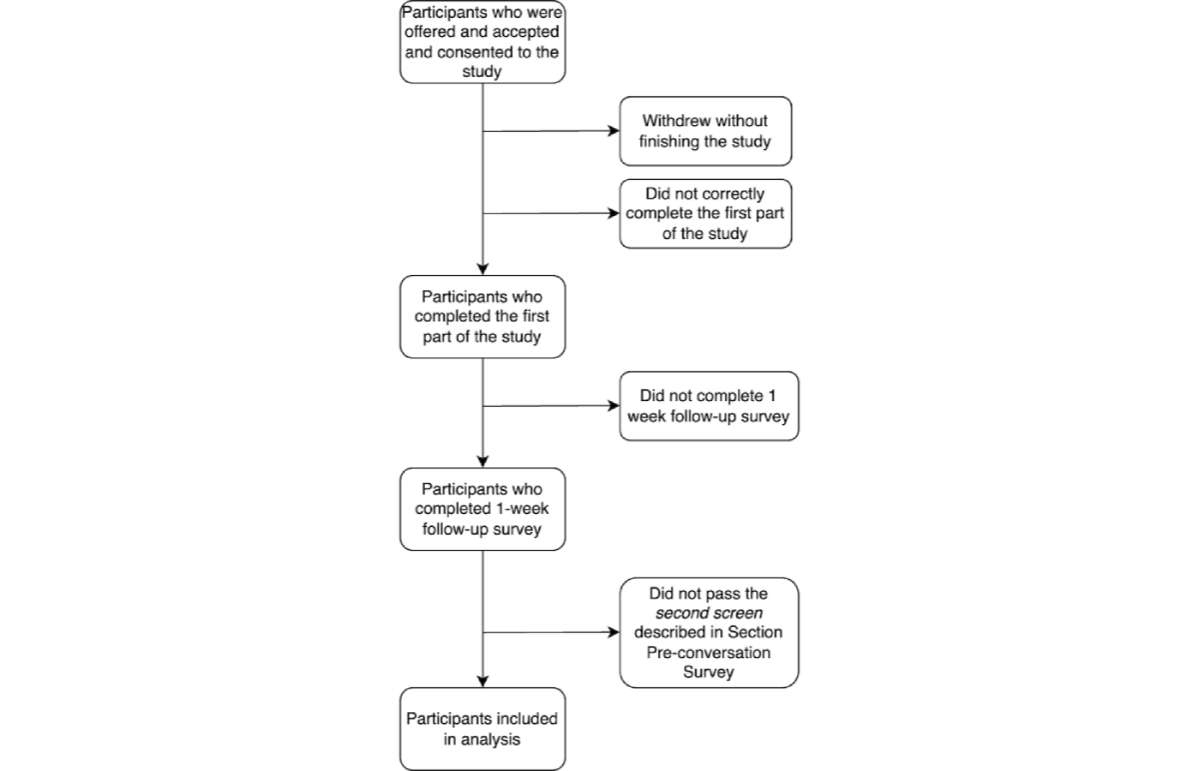
Flowchart of study procedure.

Figure 3 depicts the study procedures (also described in the *Overall Chatbot System Study Design* subsection), showing the points at which participants enter and (may) exit the study. Table 2 presents the specific exit and entry numbers for each version of the chatbot that was deployed. Of the 517 participants who completed part 1 and part 2 of the study, 168 (32.5%) were filtered out across all 4 chatbot versions using the preconversation survey criteria described in the *Preconversation Surveys and Screening* subsection, leaving 349 (67.5%) for inclusion in the analysis.

**Table 2:** Participant count for each chatbot version corresponding to the study flowchart (Figure 3).

Version	Withdrew without finishing the study, n (%)	Did not correctly complete the first part of the study, n (%)	Participants who completed the first part of the study, n (%)	Did not complete 1 week follow-up survey, n (%)	Participants who completed 1 week follow-up survey, n (%)	Did not pass the second screen described in the *Preconversation Surveys and Screening* subsection, n (%)	Values, n (%)
MIBot v4.7 (n=119)	17 (14.3)	9 (8.8)^a^	93 (91.2)^a^	0 (0)^b^	93 (100)^b^	41 (44.1)^b^	52 (55.9)^b^
MIBot v5.0 (n=171)	23 (13.5)	3 (2)^c^	145 (98)^c^	4 (2.8)^d^	141 (97.2)^d^	43 (30.5)^e^	98 (69.5)^e^
MIBot v5.1 (n=169)	24 (14.2)	4 (2.8)^f^	141 (97.2)^f^	1 (0.7)^g^	140 (99.3)^g^	41 (29.3)^h^	99 (70.7)^h^
MIBot v5.2 (n=195)	36 (18.5)	6 (3.8)^i^	153 (96.2)^i^	10 (6.5)^j^	143 (93.5)^j^	43 (30.1)^k^	100 (69.9)^k^

^a^n=102.

^b^n=93.

^c^n=148.

^d^n=145.

^e^n=141.

^f^n=145.

^g^n=141.

^h^n=140.

^i^n=159.

^j^n=153.

^k^n=143.

### HSI Survey

Table 3 provides the data from the HSI survey that was applied to the most recent versions of the chatbot (not including MIBot v4.7, for which these data were not collected).

**Table 3:** Heaviness of Smoking Index (HSI) measures.

Version	Daily number of cigarettes, mean (SD)	Time to first cigarette (answering the question “How soon after you wake up do you smoke your first cigarette?”) and count, n (%)	HSI, mean (SD)
MIBot v5.0 (n=98)	11.9 (9.3)	<5 min: 26 (26.5) 6-30 min: 15 (15.3) 31-60 min: 31 (31.6) >60 min: 26 (26.5)	1.8 (1.5)
MIBot v5.1 (n=99)	11.1 (7.9)	<5 min: 26 (26.3) 6-30 min: 27 (27.3) 31-60 min: 29 (29.3) >60 min: 17 (17.2)	1.8 (1.5)
MIBot v5.2 (n=100)	9.9 (6.1)	<5 min: 15 (15) 6-30 min: 32 (32) 31-60 min: 18 (18) >60 min: 35 (35)	1.6 (1.4)

### Participant Demographics

Multimedia Appendix 3 provides participants’ demographic data, collected by Prolific when participants enrolled on the platform. Prolific allows for these data to be revoked or changed (eg, refer to the attribute *Student Status* in Multimedia Appendix 3).

### Readiness Rulers

Participant responses to readiness rulers were collected before the chatbot conversation, immediately after, and 1 week later, as described in the *Preconversation Surveys and Screening*, *Postconversation Survey*, and *One-Week Follow-Up Survey* subsections.

Table 4 provides the means and SDs of *confidence* to quit smoking for each chatbot version before and after the conversation and 1 week later, as well as the average change after 1 week and its statistical significance.

Table 5 shows the participants’ average value for their rated *importance* to quit smoking across each experiment at each collection time, with the same format as Table 4.

Table 6 shows the participants’ average value for their rated *readiness* to quit smoking across each experiment at each collection time, with the same format as Table 4.

**Figure 4:**
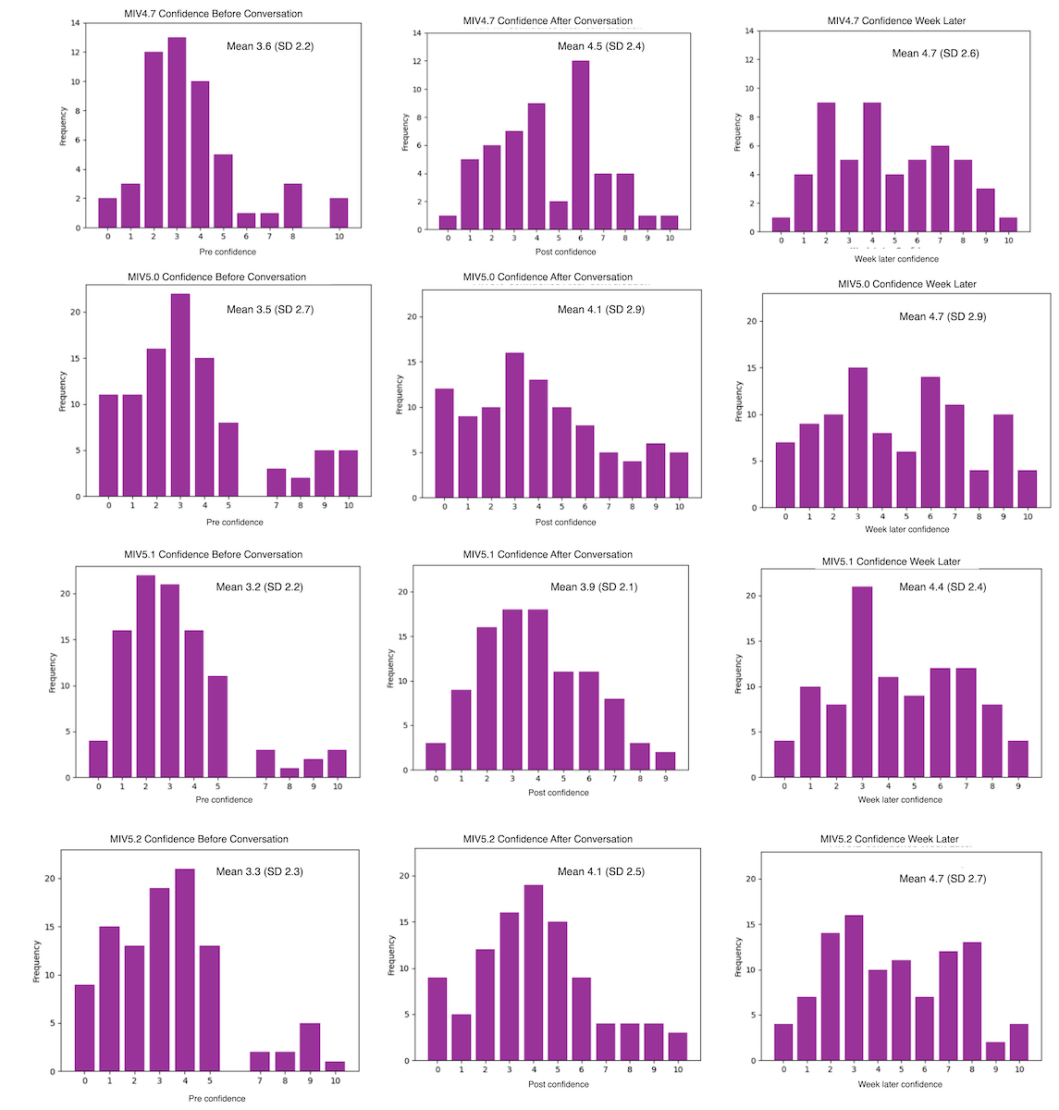
Distribution of all 4 versions’ confidence values before, after, and 1 week after the conversation.

Figure 4 provides the distribution of confidence scores in each of the 4 versions of the chatbot for each of the 3 collection points. The distributions of the importance and readiness values are provided in Multimedia Appendix 4.

Table 7 presents the number of participants whose confidence increased from before the conversation to 1 week later, the number of participants whose confidence decreased during this period, and the number of participants whose confidence stayed the same.

**Table 4:** Average confidence before, after, and 1 week after the conversation.

Version	Before the conversation, mean (SD)	After the conversation, mean (SD)	One week later, mean (SD)	Change from before the conversation to 1 week later, mean (SD)	*P* value (from paired *t* test; before the conversation to 1 week later)
MIBot v4.7	3.6 (2.2)	4.5 (2.4)	4.7 (2.6)	1.0 (2.0)	<.001
MIBot v5.0	3.5 (2.7)	4.1 (2.9)	4.7 (2.9)	1.2 (2.0)	<.001
MIBot v5.1	3.2 (2.2)	3.9 (2.1)	4.4 (2.4)	1.3 (2.3)	<.001
MIBot v5.2	3.3 (2.3)	4.1 (2.5)	4.7 (2.7)	1.3 (2.0)	<.001

**Table 5:** Average importance before, after, and 1 week after the conversation.

Version	Before the conversation, mean (SD)	After the conversation, mean (SD)	One week later, mean (SD)	Change from before the conversation to 1 week later, mean (SD)	*P* value (from paired *t* test; before the conversation to 1 week later)
MIBot v4.7	5.1 (3.1)	5.5 (3.1)	5.3 (3.1)	0.3 (1.6)	.41
MIBot v5.0	5.2 (3.0)	5.7 (3.0)	5.6 (2.8)	0.4 (1.5)	.03
MIBot v5.1	5.2 (2.8)	5.7 (2.8)	5.5 (2.9)	0.3 (1.3)	.17
MIBot v5.2	5.5 (2.9)	6.0 (2.8)	6.2 (2.8)	0.7 (2.0)	<.001

**Table 6:** Average readiness before, after, and 1 week after the conversation.

Version	Before the conversation, mean (SD)	After the conversation, mean (SD)	One week later, mean (SD)	Change from before the conversation to 1 week later, mean (SD)	*P* value (from paired *t* test; before the conversation to 1 week later)
MIBot v4.7	4.3 (2.7)	4.6 (2.6)	4.8 (2.8)	0.4 (1.5)	.09
MIBot v5.0	4.3 (2.7)	4.4 (2.8)	4.4 (2.7)	0.1 (1.8)	.75
MIBot v5.1	4.4 (2.4)	4.6 (2.4)	4.6 (2.6)	0.2 (1.5)	.14
MIBot v5.2	4.9 (2.8)	5.3 (2.7)	5.4 (2.9)	0.4 (1.7)	.01

**Table 7:** Participants whose confidence increased, decreased, or stayed the same from before the conversation to 1 week later.

Version	Confidence increased, n (%)	Confidence decreased, n (%)	Confidence stayed the same, n (%)
MIBot v4.7 (n=52)	31 (59.6)	8 (15.4)	13 (25)
MIBot v5.0 (n=98)	52 (53)	12 (12.3)	34 (34.7)
MIBot v5.1 (n=99)	50 (50.5)	21 (21.2)	28 (28.3)
MIBot v5.2 (n=100)	61 (61)	16 (16)	23 (23)

### Quit Attempts and Reduction of Smoking

Table 8 provides the number and percentage of participants, for each chatbot version, who made at least 1 quit attempt (defined as going 24 hours without smoking a cigarette) in the week before engaging in the conversation and in the week after the conversation. It should be noted that our evaluation of MIBot v4.7 did not include a survey for the number of quit attempts before the conversation.

Table 9 shows the count of participants who, after talking to the chatbot, reduced the number of cigarettes they smoke as well as those who did not reduce the number of cigarettes they smoke. The binary result of *reduce/did not reduce* was determined by setting the result to *reduce* if any one of the conditions listed in Figure S2 in Multimedia Appendix 2 was selected.

**Table 8:** Participants who made quit attempts before and after the conversation.

Version	Participants with preconversation quit attempt, n (%)	Participants with postconversation quit attempt, n (%)	*P* value (from Fisher exact test)
MIBot v4.7 (n=52)	N/A^a^	18 (35)	N/A
MIBot v5.0 (n=98)	38 (39)	33 (34)	.55
MIBot v5.1 (n=99)	26 (26)	25 (25)	.99
MIBot v5.2 (n=100)	40 (40)	38 (38)	.88

^a^N/A: not applicable.

**Table 9:** Count of participants who reduced the number of cigarettes they smoke as well as those who did not reduce the number of cigarettes they smoke.

Version	Reduced smoking after talking to the chatbot, n (%)	Did not reduce smoking after talking to the chatbot, n (%)	*P* value (from 2-sample proportion test [*z* test] against MIBot v4.7)
MIBot v4.7 (n=52)	37 (71.2)	15 (28.8)	N/A^a^
MIBot v5.0 (n=98)	68 (69.4)	30 (30.6)	.82
MIBot v5.1 (n=99)	67 (67.7)	32 (32.3)	.66
MIBot v5.2 (n=100)	74 (74)	26 (26)	.70

^a^N/A: not applicable.

### CARE Measure

Table 10 provides means and SDs for the CARE survey for each version of the chatbot as well as the results of comparisons between each version and MIBot v4.7.

**Figure 5:**
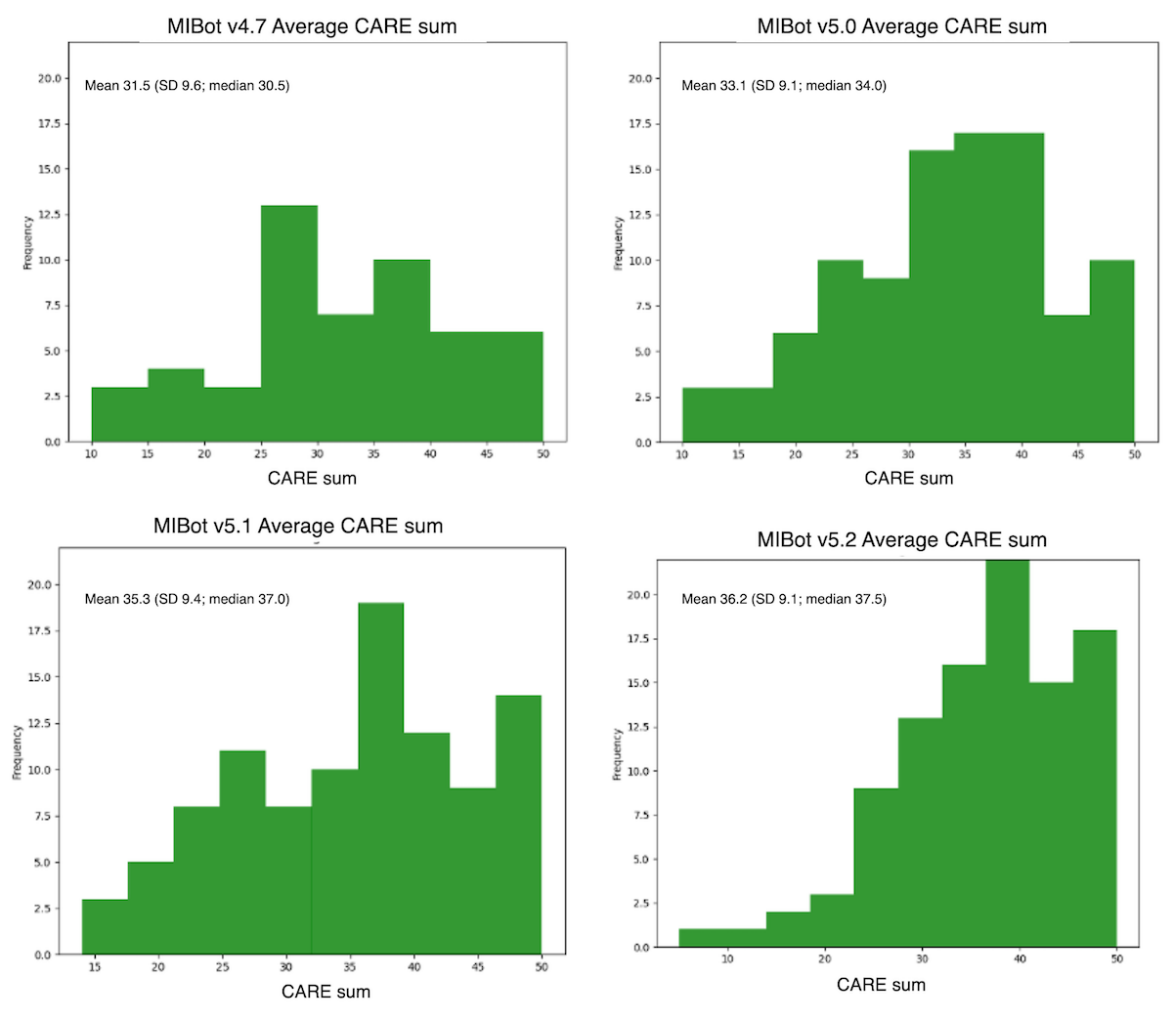
Consultation and relational empathy (CARE) survey distribution.

Figure 5 shows a histogram for each average CARE score distribution. Within each plot, the mean, median, and SD values are given.

**Table 10:** Consultation and relational empathy measure.

Version	Mean (SD)	*P* value (from the Welch *t* test against MIBot v4.7)
MIBot v4.7	31.5 (9.6)	N/A^a^
MIBot v5.0	33.1 (9.1)	.24
MIBot v5.1	35.3 (9.4)	.02
MIBot v5.2	36.2 (9.1)	.004

^a^N/A: not applicable.

### Did Ambivalence Change and in What Direction?

Table 11 presents the counts of participants who were classified as (1) moving in the direction toward quitting, (2) moving toward smoking, or (3) staying the same, as described in the *Determining Whether Ambivalence Moved Toward Quitting or Smoking* subsection. Across all 4 chatbot versions, none of the values in each class were statistically significant. Multimedia Appendix 5 provides the classification for each participant and the raw data upon which the classification was based.

**Table 11:** Counts of quit, smoke, and same ambivalence classes.

Version	Toward Quit class, n (%)	Toward Smoke class, n (%)	Stayed Same class, n (%)
MIBot v4.7 (n=52)	17 (32.7)	2 (3.8)	33 (63.5)
MIBot v5.0 (n=98)	26 (26.5)	1 (1)	71 (72.4)
MIBot v5.1 (n=99)	20 (20.2)	4 (4)	75 (75.8)
MIBot v5.2 (n=100)	30 (30)	6 (6)	64 (64)

## Discussion

### Principal Findings

The long-term goal of this work is to evolve a chatbot to have an impact on a smoker’s readiness to quit smoking, with a focus in this study on whether generative reflections can improve chatbot efficacy. Although the readiness ruler measures 3 attributes—confidence, importance, and readiness—the confidence measure (which relates to self-efficacy) most successfully predicts quitting success. The more confident someone is, the more likely they are to make a quit attempt and succeed [45-47]. Table 4 shows that all 4 versions of the conversation achieved a statistically significant improvement in confidence 1 week after the conversation took place. The average increase in confidence ranged from 1 to 1.3 on an 11-point scale. This finding is consistent with that of He et al [19] who found that a short chatbot intervention about smoking cessation can have a significant impact on quitting intentions and behaviors. In addition, although the average increase is greater for the later versions (MIBot v5.0 through MIBot v5.2), these are not statistically significant changes between versions of the chatbot (eg, *P*=.43 for MIBot v5.2 vs MIBot v4.7).

Although we hypothesized that generative responses that are specific to what a smoker says would lead to better outcomes, this result suggests that simply asking questions is sufficient to evoke most of the impact on confidence that we observed. However, there is some evidence to suggest that the improvements to the conversation beyond MIBot v4.7 (ie, generative reflections and extended dialogue) had a positive impact on participants’ readiness to quit with respect to increases in importance and readiness (refer to Tables 5 and 6, which show that MIBot v5.2 is the only version associated with significant increases in these attributes). In addition, the perceived empathy of MIBot v5.2 is significantly greater than that of MIBot v4.7 (Table 10). This makes intuitive sense because a response that addresses what a person says should be perceived as more empathetic than a response such as “Thank you for answering.” Our result contrasts the finding of He et al [19] of no significant difference in perceived empathy between a chatbot that performs MI and one that does not. It is possible that the use of generative reflections (vs the scripted reflections and responses of the study by He et al [19]) is the cause of the difference.

A study by Bikker et al [48] showed that a smoking cessation conversation by human practitioners received a high score on the CARE survey, that is, 46 (with 48% of the nurses achieving a perfect score), which is much higher than the score achieved by MIBot v5.2, that is, an average of 36 (with only 3/100, 3% of the interactions receiving a perfect score). Thus, although much of the benefits for confidence in quit readiness can be attributed to simply asking MI questions, there may be other benefits related to producing generative responses and reflections for importance and readiness, as well as the perceived empathy of the chatbot. These findings are encouraging and support further evolution of this capability.

### Recruitment and Demographics

The demographic characteristics of participants in our study (Multimedia Appendix 3) notably differ from those of participants in prior MI intervention studies in 2 ways. First, their mean age (ie, 30 years) is somewhat lower than that of participants in prior studies of human-to-human MI interventions (ie, 35 years) [49]. Second, we balanced our sample of men and women, whereas in many MI studies, approximately 68% of the participants tend to be women [49]. Third, based on the HSI survey (Table 3), participants in our study tended to smoke fewer cigarettes (ie, a mean of 10.8 cigarettes daily) than participants in studies of human-to-human MI interventions (ie, 16 cigarettes on average) [49]. These findings suggest that the participants in our study are younger and overall lighter smokers than those in typical MI studies.

Figure 3 and Table 2 show the number of participants entering each study and the number of exits from the study. The last but one column (“Did not pass the second screen described in the *Preconversation Surveys and Screening* subsection”) of Table 2 shows that many of the participants (168/349, 48.1%) did not meet the secondary screening criteria: that they were confident that they could quit smoking and thought that doing so was important. Carpenter et al [50] show that, globally, 20% of smokers are in a similar state, already motivated to quit. It may be that the younger demographic of this study accounts for this difference.

### Quit Attempts and Reduction of Smoking

The number of quit attempts related to interacting with each version of the chatbot did not significantly change from the week before the conversation to the week after (Table 8). However, the percentages of participants who attempted to quit across all versions were in the 25% (25/99) to 40% (40/100) range, much higher than the 11% that has been reported occurring 4 to 8 weeks after human MI interventions [49]. This difference in quit attempts may be related to the demographic differences we observed in our sample compared with those in other MI studies. We speculate that the groups in our studies were more likely to make quit attempts because they are a younger and less addicted population, as discussed in the *Recruitment and Demographics* subsection.

Across all conversations, Table 9 shows that a large fraction of the participants (246/349, 70.5%) did make some kind of smoking reduction attempt—meaning that they checked one of the boxes in Figure S3 in Multimedia Appendix 2. However, the differences in percentages were not significant among the different groups and chatbot versions.

### Resolution of Ambivalence

We used an alternative measure of the chatbot’s impact by classifying participants, based on their ambivalence status, as moving toward quitting, moving toward smoking, or staying the same. There was no significant difference among the chatbot versions in the percentage of participants belonging to each category (Table 11).

It is possible that the participants who resolved their ambivalence toward quitting were just ready to do this and were going to do it anyway or that the conversation was just the push they needed to go there.

It is important to consider the possibility that the 3.7% (13/349) of the participants who resolved their ambivalence to continue smoking were hurt by the interaction with the chatbot. We manually reviewed each of these conversations, and for 85.1% (297/349) of the conversations, we did not see evidence of harmful statements made by the chatbot that could have contributed to this resolution. For the other 14.9% (52/349) of the conversations, the chatbot produced poor reflections, which may have caused participants to be less likely to quit or to believe that they had less of a chance to do so; for example, in response to a participant expressing the idea that quitting *cold turkey* was their best approach to quitting, the chatbot responded, “A smoker can’t really do that,” which is quite inappropriate.

### Limitations

Our findings should be considered in the context of several limitations. First, the self-reported measures used in our study to evaluate the various chatbot versions (ie, readiness ruler, HSI survey, CARE measure, self-reported cigarette consumption, and change in smoking behavior) are potentially less accurate than a clinician-administered survey. Research suggests that participants in health studies tend to underreport unhealthy behaviors and overreport intentions to improve [51]. This tendency may account for some differences in smoking behaviors observed between our sample and other MI studies (eg, quit attempts). In addition, the data suggested that the metric *number of quit attempts* was interpreted differently by different participants; therefore this metric is not particularly reliable.

Furthermore, participants were informed that the aim of the conversation was to help improve the chatbot, which may have led them to respond in what they believed to be a desirable way after the conversation, rather than expressing their true feelings. Participants recruited in this study were also financially compensated, contingent on a review of their responses, which may have led them to agree with statements on the surveys even when they disagreed (ie, acquiescence bias [52]). Although such tendencies would apply to all chatbot versions (and not apply to comparisons among them), they limit the conclusions drawn about pre- to postconversation comparisons. Nevertheless, 1 purpose of the survey administered 1 week later was to give participants time to forget their answers to the initial surveys and to see whether the impact persists over time.

Of the 654 participants who accepted and consented to the study, 105 (16.1%) did not finish the entire study. We speculate that this dropout was caused by several factors: some of the participants may have realized that they were unwilling to discuss their smoking addiction, whereas others may have encountered technical difficulties or became distracted by other tasks because they worked from their own homes.

There is also variance in characteristics among the populations in our chatbot versions. This effect is known as the cohort effect [53] and can be seen in Tables 5 and 6 where we see variation in participant starting values on the readiness rulers. Each population sample has different characteristics and thus has different starting values. This makes comparison among studies difficult because we lose relative significance. In this study, the iterative nature of the chatbot motivated evaluations at different temporal periods. However, to draw appropriate conclusions about the impacts of different versions, future research should randomize smokers to interact with one of the various versions, or with a control in a randomized controlled trial, to eliminate such cohort effects.

### Conclusions

In this paper, we have presented a scientific and engineering framework for measuring the effect of an automated conversation on a smoker’s readiness to quit smoking. Using this framework, we evaluated how 4 versions of the conversation affected this readiness. We found that simply asking relevant questions about smoking was sufficient to confer benefits on the confidence attribute of the readiness ruler, whereas generated reflections may additionally increase other readiness attributes, while making the chatbot appear more empathetic.

Moving forward, we propose 2 avenues for future progress. First, we intend to design a more complex, longer conversation that uses more aspects of a clinician-delivered MI conversation. We hypothesize that a longer, more nuanced interaction will help to invoke more contemplation in participants. Second, we intend to improve the quality of the reflections generated. More recent models such as GPT-3 [14], ChatGPT [26], and GPT-4 [54] have been shown to be very powerful natural language generating machines, and these capabilities may be leveraged in service of generating more effective responses and reflections, with more clinical impact. If future versions of the chatbot are capable of achieving outcomes comparable with those achieved by human-delivered MI, their widespread delivery has strong potential to help smokers quit smoking sooner, perhaps preventing illness and even loss of life.

## Appendix

Multimedia Appendix 1 Examples of the 4 versions of chatbot conversation.

Multimedia Appendix 2 Chatbot survey screenshots.

Multimedia Appendix 3 Demographics of participants.

Multimedia Appendix 4 Importance and readiness values.

Multimedia Appendix 5 Chatbot feedback and confidence change with ambivalence resolution label.

## Abbreviations

BERT: Bidirectional Encoder Representations from Transformers
CARE: consultation and relational empathy
CPD: cigarettes per day
EC2: Elastic Compute Cloud
GPT-2: generative pretrained transformer 2
HSI: Heaviness of Smoking Index
MI: motivational interviewing
NLP: natural language processing
NLU: natural language understanding
QA: question answering
TTF: time to the first cigarette of the day
