# Supplementary material for: A Motivational Interviewing Chatbot With Generative Reflections for Increasing Readiness to Quit Smoking: Iterative Development Study
Source: JMIR Ment Health. 2023 Oct 17;10:e49132. doi: 10.2196/49132 (PMC10618902; doi:10.2196/49132)
Supplement: Multimedia Appendix 1 [file mental_v10i1e49132_app1.docx]

## Multimedia Appendix 1

## Examples of the Four Versions of the Chatbot Conversation

**MI Chatbot Version 4.7 - Questions but no reflections**

**BOT:** Hello! My name is MIBot, I am a computer chatbot and part of a research project at the University of Toronto. I was made by a group of researchers and doctors, with the goal of exploring your smoking behaviours.

**BOT:** I will ask a few questions, listen to your answers, and then respond.

**BOT:** However, I am learning how to have this conversation. So, I may ask you if the things I say make sense.

**BOT:** Would you like to chat with me about your smoking?

**USER:** yes

**BOT:** Great!

**BOT:** To start, what is the thing you like most about smoking?

**USER:** I like how it makes me feel relaxed

**BOT:** Thank you for your answer.

**BOT:** Now, what is the thing you like least about smoking?

**USER:** it being bad for my health

**BOT:** Thanks for your response.

**BOT:** Now, what is one thing about your smoking habit that you would like to change?

**USER:** being able to control better how much I actually smoke

**BOT:** Thank you for answering.

**BOT:** What will it look like when you have made this change in your smoking habit?

**USER:** I'll be healthier and waste less money on cigarettes

**BOT:** Thank you for your response.

**BOT:** Finally, what are the steps you need to take to make this change?

**USER:** be emotionally stable and less stressed overall

**BOT:** Thanks for your answer.

**BOT:** Thank you very much for this conversation!

**MI Chatbot Version 5.0 - Questions & Reflections with Generator Version 1**

**BOT:** Hello! My name is MIBot, I am a computer chatbot and part of a research project at the University of Toronto. I was made by a group of researchers and doctors, with the goal of exploring your smoking behaviours.

**BOT:** I will ask a few questions, listen to your answers, and then respond.

**BOT:** However, I am learning how to have this conversation. So, I may ask you if the things I say make sense.

**BOT:** Would you like to chat with me about your smoking?

**USER:** sure thing

**BOT:** Great!

**BOT:** To start, what is the thing you like most about smoking?

**USER:** the quiet moments

**BOT:** You feel that smoking helps you relax.

**BOT:** Did that make sense?

**USER:** yes

**BOT:** That's great to hear, thanks for letting me know!

**BOT:** Now, what is the thing you like least about smoking?

**USER:** it gives me too much motivation to keep doing it

**BOT:** You feel as if your smoking habit is holding you back.

**BOT:** Did what I said make sense to you?

**USER:** no

**BOT:** I am sorry to hear this, thank you for clarifying!

**BOT:** Now, what is one thing about your smoking habit that you would like to change?

**USER:** i'd like to be less judged by doing it

**BOT:** You don't want people to judge you for your habit.

**BOT:** Did that make sense?

**USER:** yes

**BOT:** Great, let’s continue

**BOT:** What will it look like when you have made this change in your smoking habit?

**USER:** i feel that when i do end up quitting smoking, it's out of necessity or out of caring too much about someone

**BOT:** You're worried that if you smoke, someone will be hurt or killed by the effects of your smoking.

**BOT:** Does this make sense to you?

**USER:** more or less

**BOT:** Great, this lets me know I'm on the right track

**BOT:** Finally, what are the steps you need to take to make this change?

**USER:** i guess meet the right person ?

**BOT:** It's not always easy to find the right person.

**BOT:** Did that make sense?

**USER:** yes

**BOT:** Thanks, that is good to hear!

**BOT:** Thank you very much for this conversation, it was really helpful!

**MI Chatbot Version 5.1 - Questions & Reflections with Generator Version 2**

**BOT:** Hello! My name is MIBot, I am a computer chatbot and part of a research project at the University of Toronto. I was made by a group of researchers and doctors, with the goal of exploring your smoking behaviours.

**BOT:** I will ask a few questions, listen to your answers, and then respond.

**BOT:** However, I am learning how to have this conversation. So, I may ask you if the things I say make sense.

**BOT:** Would you like to chat with me about your smoking?

**USER:** Sure.

**BOT:** Great!

**BOT:** To start, what is the thing you like most about smoking?

**USER:** stress relief

**BOT:** You enjoy the relief that smoking provides you.

**BOT:** Did that make sense?

**USER:** Yes.

**BOT:** That's great to hear, thanks for letting me know!

**BOT:** Now, what is the thing you like least about smoking?

**USER:** Health issues.

**BOT:** You are aware of the health risks associated with smoking and are concerned about your own health.

**BOT:** Did what I said make sense to you?

**USER:** Yes.

**BOT:** Thanks, I'm glad, this helps my listening skills

**BOT:** Now, what is one thing about your smoking habit that you would like to change?

**USER:** Smoking among friends.

**BOT:** You would like to stop smoking among your friends.

**BOT:** Did that make sense?

**USER:** Yeah.

**BOT:** Great, let’s continue

**BOT:** What will it look like when you have made this change in your smoking habit?

**USER:** Stop smoking or smoking less

**BOT:** You will stop smoking completely.

**BOT:** Does this make sense to you?

**USER:** Yes.

**BOT:** Great, this lets me know I'm on the right track

**BOT:** Finally, what are the steps you need to take to make this change?

**USER:** Taking nicotine tablets.

**BOT:** You realize that you need to replace smoking with nicotine replacement therapy (NRT).

**BOT:** Did that make sense?

**USER:** Yea

**BOT:** Thanks, that is good to hear!

**BOT:** Thank you very much for this conversation, it was really helpful!

**MI Chatbot Version 5.2 - Enhanced/Extended Conversation based on v5.1**

**BOT:** Hello! My name is MIBot, I am a computer chatbot and part of a research project at the University of Toronto. I was made by a group of researchers and doctors, with the goal of exploring your smoking behaviours.

**BOT:** I will ask a few questions, listen to your answers, and then respond.

**BOT:** However, I am learning how to have this conversation. So, I may ask you if the things I say make sense.

**BOT:** Would you like to chat with me about your smoking?

**USER:** Yes please

**BOT:** Great!

**BOT:** To start, what is the thing you like most about smoking?

**USER:** stress relief

**BOT:** You enjoy smoking because it helps you cope with stressful situations.

**BOT:** Did that make sense?

**USER:** yes

**BOT:** That's great to hear, thanks for letting me know!

**BOT:** What else do you like about smoking?

**USER:** alone time

**BOT:** You enjoy spending time by yourself with cigarettes.

**BOT:** Did that make sense?

**USER:** yes

**BOT:** That's great to hear, thanks for letting me know!

**BOT:** Now, what is the thing you like least about smoking?

**USER:** the money

**BOT:** You dislike spending money on cigarettes.

**BOT:** Did what I said make sense to you?

**USER:** yes

**BOT:** That's great to hear, thanks for letting me know!

**BOT:** What else do you dislike about smoking?

**USER:** the addiction

**BOT:** You are aware that cigarettes can be addictive and you worry about how much you smoke.

**BOT:** Did that make sense?

**USER:** yes

**BOT:** Thanks, I'm glad, this helps my listening skills

**BOT:** Now, what is one thing about your smoking addiction that you would like to change?

**USER:** the amount I smoke

**BOT:** You want to reduce the amount of cigarettes you smoke.

**BOT:** Did that make sense?

**USER:** yes

**BOT:** Thanks, that is good to hear!

**BOT:** It's great to hear you want to reduce your smoking. What would it look like when you have reduced your smoking addiction?

**USER:** id be smoking 10 cigs per day

**BOT:** You'd smoke less than 10 cigarettes per day.

**USER:** yes

**BOT:** Understood, thank you for sharing.

**BOT:** What might be helpful towards reducing your smoking?

**USER:** not sure

**BOT:** You are unsure about what your life will look like after making this change.

**BOT:** Did that make sense?

**USER:** yes

**BOT:** Great, this lets me know I'm on the right track

**BOT:** Finally, what are the steps you need to take to make this change?

**USER:** try and reduce by one cig per day

**BOT:** You want to reduce your smoking by one cigarette per day.

**BOT:** Did that make sense?

**USER:** yes

**BOT:** Thanks, that is good to hear!

**BOT:** Thank you very much for this conversation, it was really helpful!
