# Supplementary material for: A Motivational Interviewing Chatbot With Generative Reflections for Increasing Readiness to Quit Smoking: Iterative Development Study
Source: JMIR Ment Health. 2023 Oct 17;10:e49132. doi: 10.2196/49132 (PMC10618902; doi:10.2196/49132)
Supplement: Multimedia Appendix 2 [file mental_v10i1e49132_app2.docx]

## Multimedia Appendix 2

## Chatbot Survey Screenshots


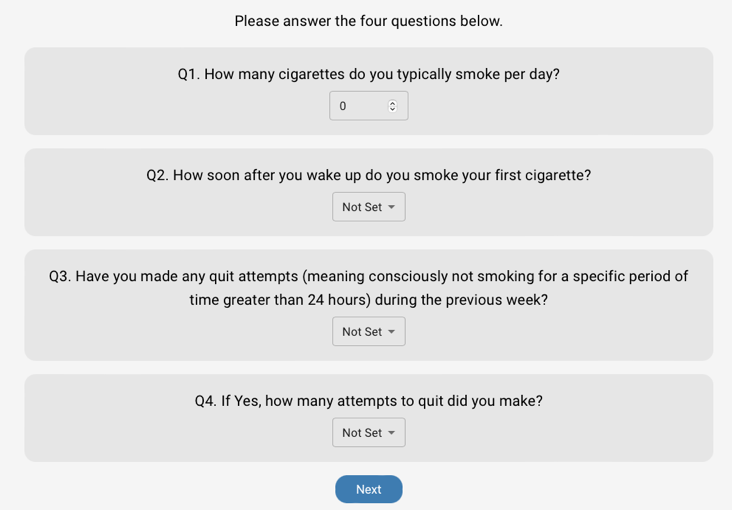


Figure S1 - Screen that measures HSI and Quit Attempts


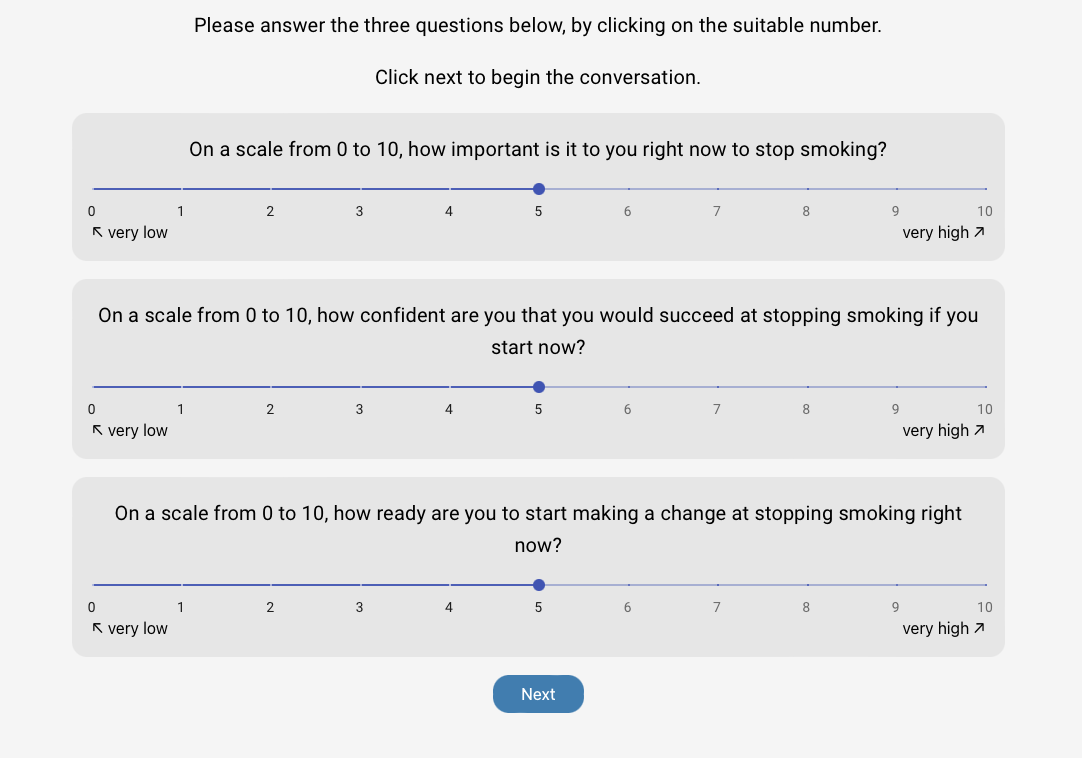


Figure S2 - The Readiness Ruler


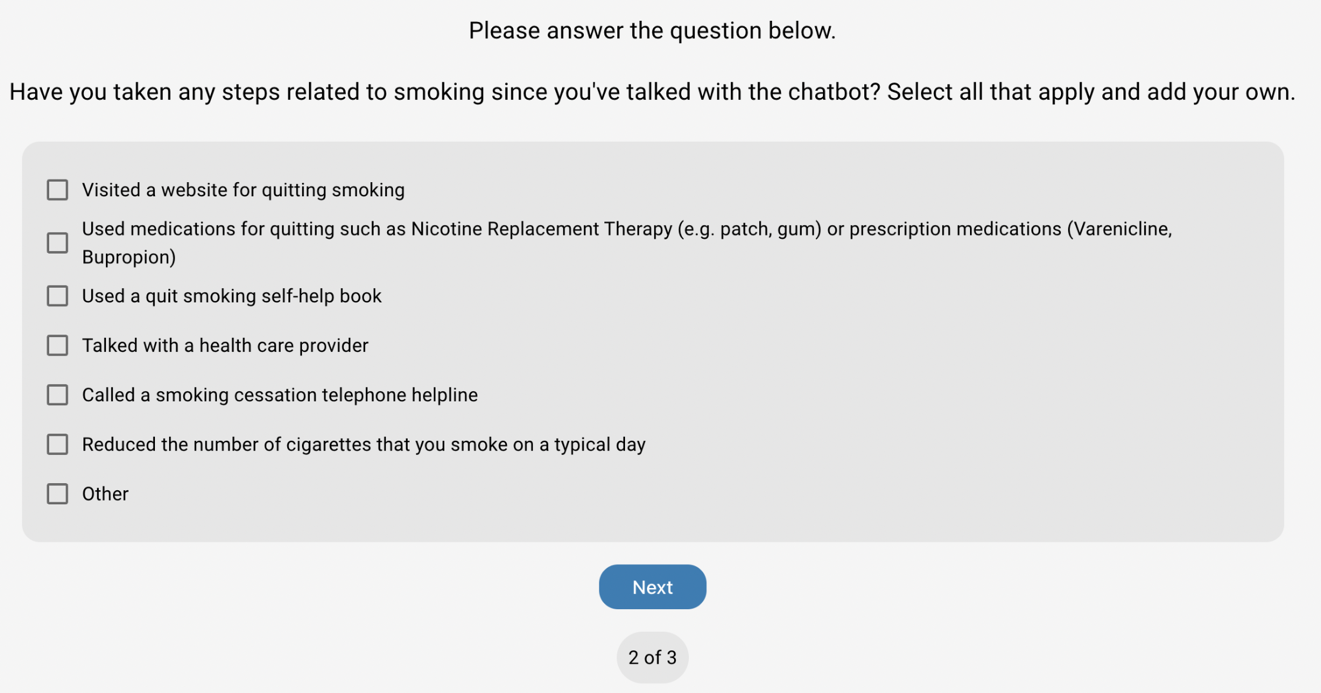


Figure S3 – One Week Later Quit Smoking Actions
