# Supplementary material for: A Motivational Interviewing Chatbot With Generative Reflections for Increasing Readiness to Quit Smoking: Iterative Development Study
Source: JMIR Ment Health. 2023 Oct 17;10:e49132. doi: 10.2196/49132 (PMC10618902; doi:10.2196/49132)
Supplement: Multimedia Appendix 3 [file mental_v10i1e49132_app3.docx]

## Multimedia Appendix 3

## Demographics of Participants

| **Demographic Factors** Count, n (%) | **MIV4.7** | **MIV5.0** | **MIV5.1** | **MIV5.2** |
| --- | --- | --- | --- | --- |
| **Sex** |  |  |  |  |
| Female | 30 (57.7) | 46 (47) | 54 (54.5) | 51 (51) |
| Male | 22 (42.3) | 52 (53) | 45 (45.5) | 49 (49) |
| **Age** |  |  |  |  |
| 18 to 19 | 2 (3.8) | 5 (5.1) | 1 (1) | 2 (2) |
| 20 to 29 | 35 (67.3) | 58 (59.2) | 50 (50.5) | 58 (58) |
| 30 to 39 | 8 (15.5) | 16 (16.3) | 22 (22.2) | 26 (26) |
| 40 to 49 | 5 (9.6) | 14 (14.3) | 12 (12.1) | 10 (10) |
| 50 to 59 | 2 (3.8) | 5 (5.1) | 11 (11.1) | 3 (3) |
| ≥ 60 | 0 (0) | 0 | 3 (3.1) | 1 (1) |
| **Student Status** |  |  |  |  |
| Yes | 27 (51.9) | 45 (45.9) | 32 (32.3) | 48 (48) |
| No | 25 (48.1) | 52 (53.1) | 59 (59.6) | 45 (45) |
| Data Revoked | 0 (0) | 1 (1) | 8 (8.1) | 7 (7) |
| **Employment Status** |  |  |  |  |
| Full-Time | 23 (44.2) | 39 (39.8) | 42 (45.2) | 41 (41) |
| Part-Time | 10 (19.2) | 22 (22.5) | 16 (17.2) | 19 (19) |
| Unemployed (and job seeking) | 9 (17.4) | 19 (19.4) | 16 (17.2) | 21 (21) |
| Not Paid in Work | 5 (9.6) | 6 (6.1) | 12 (12.9) | 0 (0) |
| Other | 5 (9.6) | 12 (12.2) | 7 (7.5) | 19 (19) |
| **Average Total Approvals (for all participant studies)** | 233.8 | 262.5 | 360.8 | 254.8 |
| **Average Approval Rate (for all participant studies)** | 99% | 99% | 99% | ~ |
