# Supplementary figures and images for: A Motivational Interviewing Chatbot With Generative Reflections for Increasing Readiness to Quit Smoking: Iterative Development Study
Source: JMIR Ment Health. 2023 Oct 17;10:e49132. doi: 10.2196/49132 (PMC10618902; doi:10.2196/49132)

## Multimedia Appendix 4

## Importance and Readiness Values

**
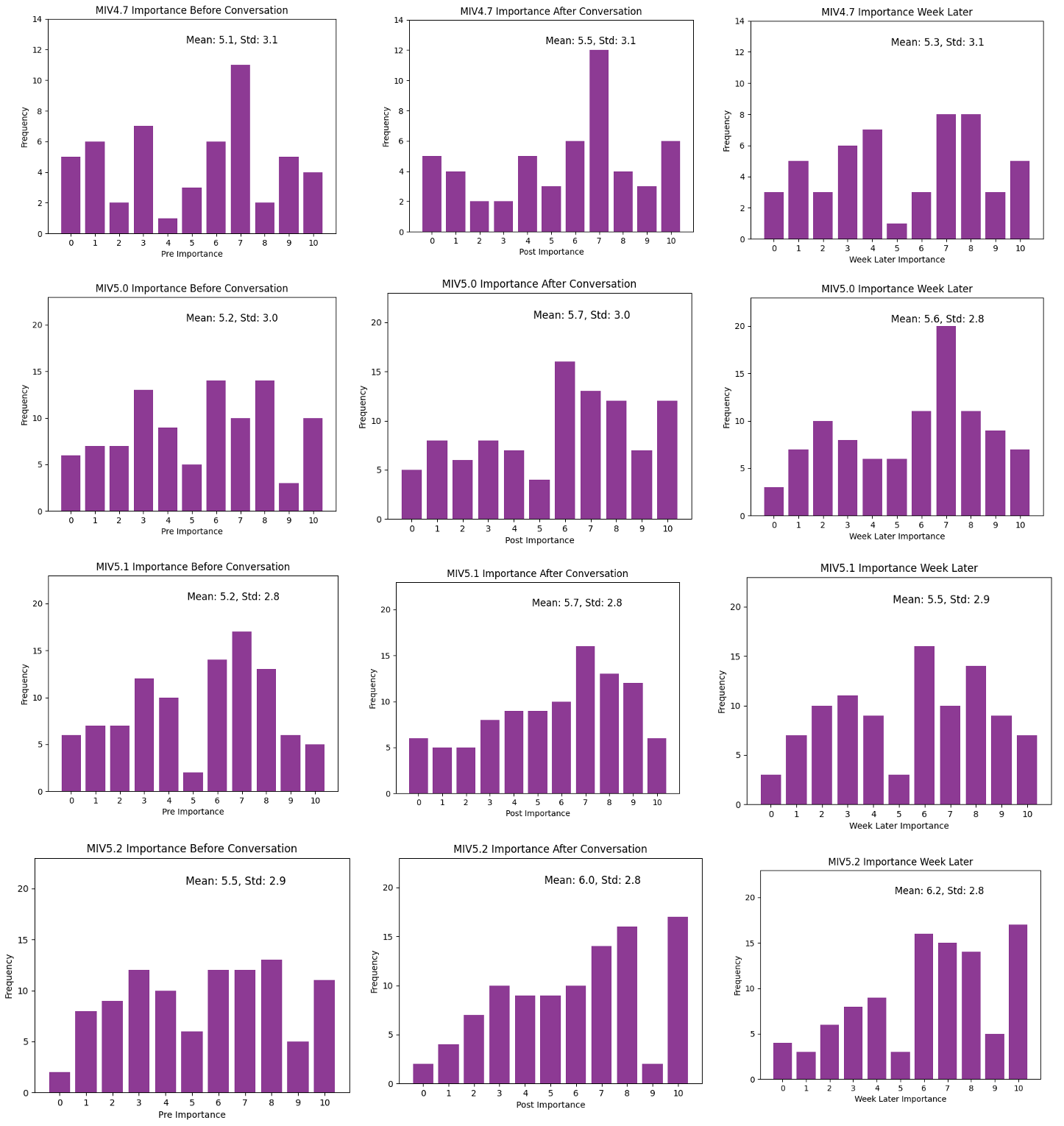
**


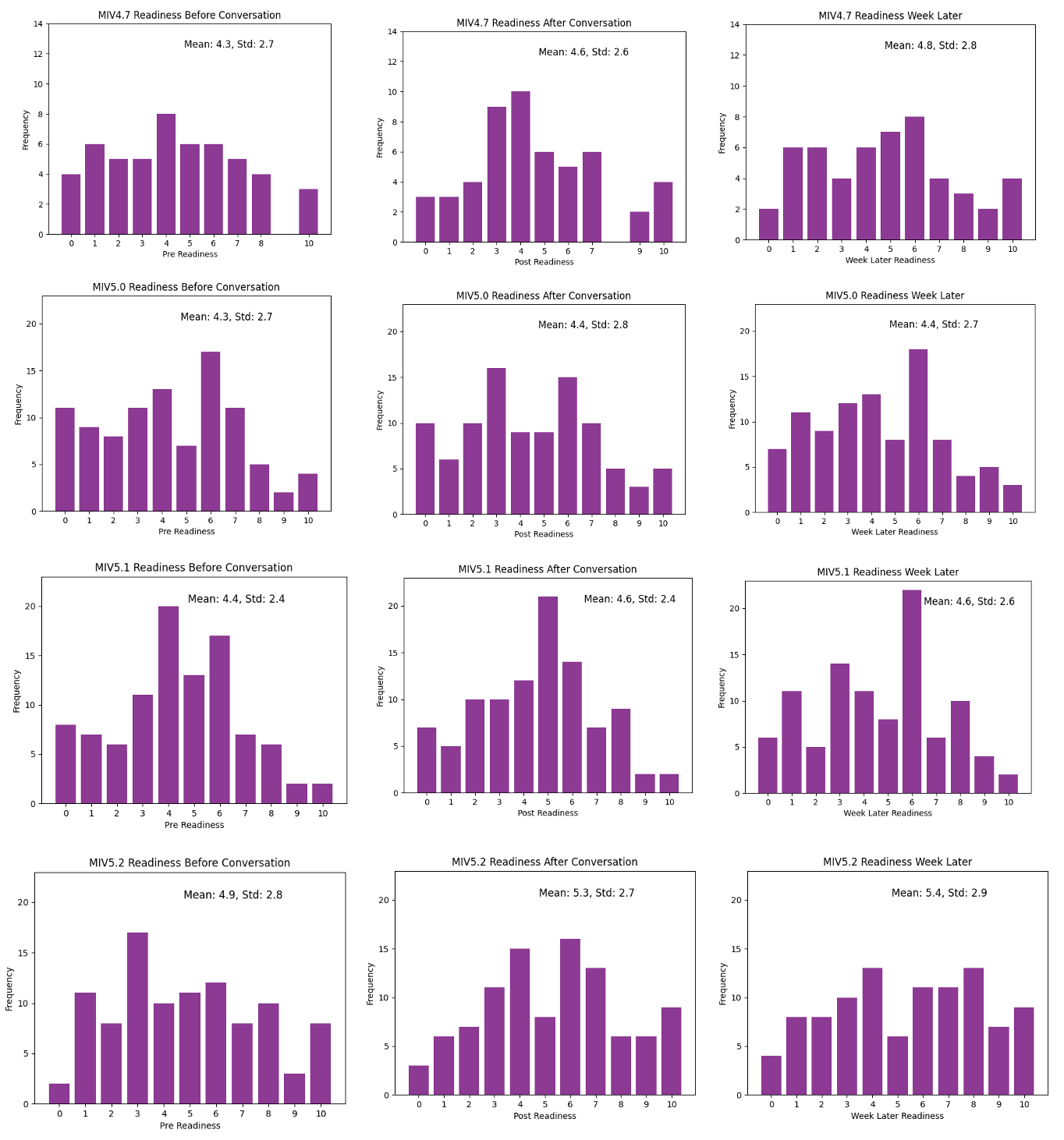

Supplement: Multimedia Appendix 4 [file mental_v10i1e49132_app4.docx]
