# Supplementary material for: A Motivational Interviewing Chatbot With Generative Reflections for Increasing Readiness to Quit Smoking: Iterative Development Study
Source: JMIR Ment Health. 2023 Oct 17;10:e49132. doi: 10.2196/49132 (PMC10618902; doi:10.2196/49132)
Supplement: Multimedia Appendix 5 [file mental_v10i1e49132_app5.docx]

## Multimedia Appendix 5

## Chatbot Feedback and Confidence Change with Ambivalence Resolution Label

| Experiment Name | Confidence Quit | Feedback | Final Label |
| --- | --- | --- | --- |
| MIV4.7 | -6 | Just made me realise i dont what to stop smoking right now | Smoke |
| MIV4.7 | -3 | It helped me understand that in order to Quit something one should plan and know the steps that one needs to take | Same |
| MIV4.7 | -2 | No. It was pretty fair | Same |
| MIV4.7 | -1 | It didn't because they were just normal questions and I already knew the answers | Same |
| MIV4.7 | -1 | No, because the questions asked are already things that i know and think about frequently, nothing really made me re-think my behaviour regarding smoking | Same |
| MIV4.7 | -1 | Oh yeah surerly that im addicted to it because i may hate myself internally as well as im ''used'' to it already in a way | Same |
| MIV4.7 | -1 | Not really. I already know (before the chat) that I have to smoke less. | Same |
| MIV4.7 | -1 | Not really. I am conscious about what it implies to smoke and I do it regard this. I think the conversation can help people who actually want to quit smoking, but It's not my case. | Smoke |
| MIV4.7 | 0 | Yes it did. It made me think about my behaviour and how it impacts my health | Same |
| MIV4.7 | 0 | It brought me closer to the idea that quitting smoking would do me good | Same |
| MIV4.7 | 0 | No. The questions didn't really make me think. Kinda just made me feel irritated since it's the same questions everyone asks smokers. | Same |
| MIV4.7 | 0 | Honestly no, the bot didn't do or said anything new nor remotely helpful | Same |
| MIV4.7 | 0 | Somethings I already knew, it's a topic I think about monthly at least, but yes | Same |
| MIV4.7 | 0 | no, too short and impersonal | Same |
| MIV4.7 | 0 | Not really. Felt like an automatic response. Like I was speaking to myself and Ive done that a thousand times | Same |
| MIV4.7 | 0 | Not actually, because they were basically just collecting data, but not giving me any feedback. | Same |
| MIV4.7 | 0 | It helped me to realise I probably smoke more cigarettes than I truly need to, although it didn't help in any practical way for methods on how to cut down. | Same |
| MIV4.7 | 0 | I don't really think it did. The conversation felt more like monologue, chatbot was asking questions and I was answering them. He didn't say anything interesting or new for me at all. | Same |
| MIV4.7 | 0 | Yes..because the chatbox makes me feel uncomfortable | Same |
| MIV4.7 | 0 | It did not. I was already very aware of the pros and cons that smoking brings into my life, as I have had a lot of introspection regarding that concern, in the past. | Same |
| MIV4.7 | 0 | No, every thing that I have said I already have thought about | Same |
| MIV4.7 | 1 | nope, it was just few questions | Same |
| MIV4.7 | 1 | It makes me think that maybe it is better to stop, it points out to me but it is difficult to do so | Quit |
| MIV4.7 | 1 | Not really. I know the Quits i need to make. its just hard to do them | Same |
| MIV4.7 | 1 | Acutally it did. It brought out 2 main problems I have with smoking (money & drink-smoking) | Quit |
| MIV4.7 | 1 | Yes. It made me realize that even though I am not ready to give up smoking yet, that there is in fact something I don't like about my smoking habit and that I already have the knowledge of how to Quit that. | Same |
| MIV4.7 | 1 | Yes, i need to think more about stopping smoking for my family's sake as well as my own. | Quit |
| MIV4.7 | 1 | This is how I began to analyze how much I smoke, how much I spend on cigarettes and under what conditions I smoke less or not at all. | Quit |
| MIV4.7 | 1 | Not really but only because this is something I have been aware for a while now and I think about it quite regularly but it feels like for some reason I am not willing to do it. | Same |
| MIV4.7 | 1 | nothing i didn't know before, it is a good opportunity for reflection. It helps you to think about things and say things you might avoid in fear of others expectations or failure. | Same |
| MIV4.7 | 1 | Bot only asked questions and didnt provide any solutions so no, the chatbot didnt make me realize anything about my smoking behavior | Same |
| MIV4.7 | 1 | not really | Same |
| MIV4.7 | 1 | I realized a little that I should try harder to smoke less. | Quit |
| MIV4.7 | 1 | It highlighted concerns that have been sitting in the back of my mind about my smoking behavior, it brought them to the forefront of my mind. | Quit |
| MIV4.7 | 1 | Only what I already knew: that there's no advantages that overpower the disadvantages | Same |
| MIV4.7 | 2 | Yes, it made me reflect about it and decide where to start changing my habit | Quit |
| MIV4.7 | 2 | Yes, because from the questions he asked me, I realized that I can try to quit smoking and that it is willpower. | Quit |
| MIV4.7 | 2 | The conversation makes you think about what you need to do to find the willpower to do good things for your health... | Quit |
| MIV4.7 | 2 | Not much but I would think about that | Same |
| MIV4.7 | 2 | Yes, because i had to face that im an addict | Quit |
| MIV4.7 | 2 | not really, I already feel aware of my smoking behaviors | Same |
| MIV4.7 | 2 | Not really. | Same |
| MIV4.7 | 3 | That I'm aware that I have a problem. | Quit |
| MIV4.7 | 3 | I believe a bit yes because nowadays people (including me also) don't think about the addiction so it's an excellent method (like this chat) to remind ourselves about the existing problem | Quit |
| MIV4.7 | 3 | yes it made me understand even more that wanting is power | Quit |
| MIV4.7 | 3 | yes, how bad it is and the purpose there of | Quit |
| MIV4.7 | 4 | Yes, it helps to talk to someone or the Bot about addiction | Quit |
| MIV4.7 | 4 | yes, it did because to be honest I was smoking when I received this survey | Quit |
| MIV4.7 | 4 | No , my answers were things I've already realized. | Same |
| MIV4.7 | 5 | Yes, sometimes a simple question can really put things into perspective | Quit |
| MIV4.7 | 6 | It didn't make any difference, It was more of a questionnaire I think it should have given me more reasons to help me quit smoking but I do get were it's coming from. Hopefully, I'll be able to Quit my behavior in a week. | Same |
| MIV4.7 | 7 | Nothing more than I already knew | Same |
| MIV5.0 | -4 | I hadn't questioned why i've been chain smoking lately and it might me confront that. | Same |
| MIV5.0 | -3 | It did. It made me think about my smoking behavior and I think I'm going to spend more time on wondering how to stop smoking. | Same |
| MIV5.0 | -3 | Yes i need to stop and i need to start now | Same |
| MIV5.0 | -2 | It made me realise that like smoking for some reasons and hate it for others. It all comes down on my own priorites | Same |
| MIV5.0 | -2 | No, because I'm fully aware of my smoking behavior/habit | Same |
| MIV5.0 | -1 | Yes and no. I already pretty much knew everything that I spoke about. It just was funny me answering aloud (via text) what I keep in the back recesses of my mind mostly. | Same |
| MIV5.0 | -1 | Not a lot ,Just a reminder that is a bad habit and do a lot of harm , but there isn t a new info | Same |
| MIV5.0 | -1 | Not really. I know is not the healthiest thing, but is not something that I'm looking to Quit right now. I feel like there are other things more important to work on in my life. | Smoke |
| MIV5.0 | -1 | I realized what's my least favorite part in my habit, thanks to some specific questions about it. | Same |
| MIV5.0 | -1 | no, i already know | Same |
| MIV5.0 | -1 | No, in the sense that I was already aware of the answers to the questions asked regarding motivation etc | Same |
| MIV5.0 | -1 | It made me realize I haven't really thought a lot about the reasons why I smoke besides that is something that I have been doing for years. Also, it kinda make me feel bad when it refered to my smoking as "your problem" or something like that. It was like I DON'T HAVE A PROBLEM. But maybe I do? | Same |
| MIV5.0 | 0 | Yes. By asking what I liked the most about smoking and what I less liked, it made be think consider if what I like the most is more important than what I like less. | Same |
| MIV5.0 | 0 | I didn't help me really, but it opened my eyes on how easily i could get help quitting if i really wanted to | Same |
| MIV5.0 | 0 | It made me realize that I already know some of the steps needed to quit. Now it is up to me to follow through. | Same |
| MIV5.0 | 0 | It didn't help me understand anything better because it was only trying to get me to stop smoking, not actually being introspective at all. The questions were extremely open-ended and it struggled to understand complex sentences anyway, and since people are complex it's pretty essential that it can understand things. If its purpose is to simply reframe what somebody says into a biased "oh so you're trying to stop for this reason right!!!?!?!???1!!!" then it will never reveal anything the person doesn't already know about themselves. | Same |
| MIV5.0 | 0 | Not really, it would've felt better to know i was speaking to a real person. Maybe changing the name of the bot could help a little bit. | Same |
| MIV5.0 | 0 | Kind of, it helped my question myself on why do i actually smoke, what makes me want a cigarette | Same |
| MIV5.0 | 0 | yes, that I can Quit my smoking habit if I really want to. | Same |
| MIV5.0 | 0 | no I can't really say I realized anything | Same |
| MIV5.0 | 0 | It helped me reafirm that I'm not ready to quit somking because when the chatbot asked me if I knew where to begin changing my habits, I didn't know how to answer. | Same |
| MIV5.0 | 0 | I don't think it did because sometimes the chatbot didnt understood what i meant but maybe that was my problem maybe cuz i wasnt clear sometimes | Same |
| MIV5.0 | 0 | No, I still feel the same. | Same |
| MIV5.0 | 0 | In the moment yes, but i think quitting smoking is a longer process in your mind than the conversation i just had with the lovely chatbot. Again the question raised were interesting and made me reflect on my cigarette count, but that is something i already think about on the daily and try to manage | Same |
| MIV5.0 | 0 | no, because i was aware of my smoking behavior before hand. | Same |
| MIV5.0 | 0 | It didn't | Same |
| MIV5.0 | 0 | not really but that's because i already interiorized the faults in my smoking behaviour | Same |
| MIV5.0 | 0 | Yes, because I know its not a good habit | Same |
| MIV5.0 | 0 | No, the questions were mostly things that i have thought about before | Same |
| MIV5.0 | 0 | It made me realise that if I really wanted to, I could perhaps substantially reduce the frequency of my smoking simply by cutting out every other cigarette. | Same |
| MIV5.0 | 0 | No, these are things I've already considered. | Same |
| MIV5.0 | 0 | it as not, i have wanting to stop smoking a long time ago but i can never do it | Same |
| MIV5.0 | 0 | yes | Same |
| MIV5.0 | 0 | Not really, as I mentioned in the previous answer it would just point out what I described myself using more words. But on the other hand, it felt good in a weird way admitting my concerns to someone-something about smoking. | Same |
| MIV5.0 | 0 | Not really, it didn't give me any new information. | Same |
| MIV5.0 | 0 | No, because, while it made me reflect on it, I didn't find out anything surprising | Same |
| MIV5.0 | 0 | It made me think about why I smoked and why I would want to give up. | Same |
| MIV5.0 | 0 | Yes. It reminded me why I want to quit. | Same |
| MIV5.0 | 0 | No. It only gave him information, it didn't provide much for me | Same |
| MIV5.0 | 0 | not really, just highlights what i already know | Same |
| MIV5.0 | 0 | The questions were rather very simple and typical, nothing exceptional. All I could write was what I already know and become more aware of the problem. This is also important. | Same |
| MIV5.0 | 0 | No, I already had those conclusions in mind | Same |
| MIV5.0 | 0 | Not really, it just listened to everything i said, just that | Same |
| MIV5.0 | 0 | no, because of the reason I've just written. also: there's nothing to realize about smoking. we know it's costly, we know it's damaging our health. you won't find a time traveler from the 20es that will go "oh wow, what are you saying??? smoking is actually BAD for you??!?" | Same |
| MIV5.0 | 0 | it did not Quit anything. I am aware that smoking is bad for my health and I would quit it if I wanted to. I quit it for a year about 2 years ago and I could do it again only if I wanted to. | Same |
| MIV5.0 | 0 | No. Because I’m the type of user who doesn’t Quit her opinion on smoking after a random conversation, be it online or in real life | Same |
| MIV5.0 | 1 | no. i already know the problem i have | Same |
| MIV5.0 | 1 | Nothing precise. Only that I don't want to quit smoking right now. | Same |
| MIV5.0 | 1 | it did, that i sohuld seek someone i care for so much im willing to stop smoking jsut so i can life better with that person, which at the end of the day is a good food for thought, thanks | Quit |
| MIV5.0 | 1 | no, it was to basic | Same |
| MIV5.0 | 1 | No. It was a real short conversations, and I feel the bot did not understand me very well. | Same |
| MIV5.0 | 1 | yes, that my smoking habit is linked with social events and conforming wiyh my peers | Quit |
| MIV5.0 | 1 | yes, because analyze what could be the reasons why it would be worth quitting than health and expenses | Quit |
| MIV5.0 | 1 | no, didnt offer any solutions/methods | Same |
| MIV5.0 | 1 | I just reminded myself to cut down the number of cigarettes I consume daily. | Quit |
| MIV5.0 | 1 | not particularly, stressful situations do bring up my anxiety which does make me smoke more often | Quit |
| MIV5.0 | 1 | Yes it did. Namely what I like and don't like about smoking. | Quit |
| MIV5.0 | 1 | Yes, it did not mention anything specific but the fact that it repeated what I was saying in a different manner really brought my goals to my attenttion | Quit |
| MIV5.0 | 1 | No because i already know what's wrong with my smoking problem. Bot didn't tell me anything innovative | Same |
| MIV5.0 | 1 | yes it did ,because it helped me realize and remember most importantly the bad thing smoking does to my health it putted from unconscious to conscious | Quit |
| MIV5.0 | 1 | yes, similar suggestions | Quit |
| MIV5.0 | 1 | I think it kinda made me understand that if I met with people who don't smoke as much I would not be smoking as much either | Quit |
| MIV5.0 | 1 | No - it made it clear I had no clue where to start | Same |
| MIV5.0 | 1 | Not so much, as I have been going over these things in my mind lately | Same |
| MIV5.0 | 1 | It did. While thinking about the answers to the questions, i realised that my smoking is problematic for me. | Quit |
| MIV5.0 | 2 | Yes, i understand that i know what i have to do. I'm just lazy | Quit |
| MIV5.0 | 2 | Yes it made me realize that I need to quit smoking and find better ways to cope with life | Quit |
| MIV5.0 | 2 | Yes, it reminded me of how unhealthy smoking is to me | Quit |
| MIV5.0 | 2 | yes | Same |
| MIV5.0 | 2 | It did because I'm actively talking about the issue in itself but the convo in hand didn't help much in that regards. (as i mentioned in the previous answer) | Same |
| MIV5.0 | 2 | No, the responses weren't in depth enough | Same |
| MIV5.0 | 2 | No, I already understood why I smoke, for the robot I just had to formulate it | Same |
| MIV5.0 | 2 | Not really. I think I had the information in my head already and the bot just gives you a means to reflect on it. | Same |
| MIV5.0 | 2 | Yes, I need to start changing my habits. I need to stop smoking or work towards reducing my intake. | Quit |
| MIV5.0 | 2 | Not really, but had very optimistic and enthusiastic responses which made me feel a little more optimistic to quiting | Same |
| MIV5.0 | 2 | YES, MADE BE MORE AWARE ABOUT MY SMOKING HABIT | Quit |
| MIV5.0 | 3 | It actually made me realise I can quit smoking if I am determined enough because I really want to be able to participate in activities I enjoy. | Quit |
| MIV5.0 | 3 | It wasn't an enlightening chat, honestly | Same |
| MIV5.0 | 3 | nothing especially new but yes, I really need to use my "fake cigarette" which is really the best thing I ever tried till now to give up smoking; be always motivated and not only time to time - I don't like this character in other people but, finally, regarding giving up smoking, I am exactly the same! I am almost disgusted by that discovery | Quit |
| MIV5.0 | 3 | That I need to stop smoking | Quit |
| MIV5.0 | 3 | not really. it didnt give me any tips | Same |
| MIV5.0 | 3 | Yes, because I have never asked myself those questions with the intention of changing my habit. | Quit |
| MIV5.0 | 4 | Yes. It made me realise that I need to find a substitute for smoking that doesn't affect my health negatively. | Quit |
| MIV5.0 | 4 | Not really, I was already aware that I'm choosing to behave in a way that won't benefit me in the future and I already knew what are the reasons behind my habit. | Same |
| MIV5.0 | 4 | Not that much becouse I know I can't do it | Same |
| MIV5.0 | 4 | Yes, because I really want to quit smoking | Quit |
| MIV5.0 | 4 | i don't know, maybe a little bit | Same |
| MIV5.0 | 4 | Totally. The answers that I gave to the bot were not concise and didn't have any meaningful background to me, but after reading the suggestions I started thinking that those suggestions were a kind of thought that I had but at this moment I hadn't had. | Quit |
| MIV5.0 | 4 | Not really. The bot seemed to just summarize what I was saying to it. This is impressive on a technical level, but it didn't offer me anything constructive with regards to my habit. | Same |
| MIV5.0 | 4 | Not much | Same |
| MIV5.0 | 4 | Yes, i realised i smoke a lot | Quit |
| MIV5.0 | 5 | yes, it makes it real , you have to see the real problem | Same |
| MIV5.0 | 5 | It reminded me that I should keep going because I still smoke too much | Same |
| MIV5.0 | 6 | No | Same |
| MIV5.0 | 6 | Not much, I told things that I knew before. | Same |
| MIV5.0 | 6 | In part, yes, although the chatbot did not try to motivate me, but asked questions. On the other hand, this conversation made me realize that I could do it. | Quit |
| MIV5.0 | 7 | I realize that i can saves a lot of money if i start to smoke less | Quit |
| MIV5.0 | 9 | No not really, I already know smoking is horrible for me | Same |
| MIV5.1 | -7 | not at all. i love smoking, but was funny | Same |
| MIV5.1 | -3 | not really, i knew all that already | Same |
| MIV5.1 | -2 | No, because everyting about this conversation is on mind since ever | Same |
| MIV5.1 | -2 | Nothing new that I didn't know before. I'm aware of the fact that most of the cigarettes in the week aren't enjoyable. But I'm smoking because 1 of 10 feels good, especially at the parties etc. | Smoke |
| MIV5.1 | -2 | Yes, for a long time I didn't have conversation about my smoking habits and how many cigarettes I smoke daily. | Same |
| MIV5.1 | -2 | No. It's just a personal decision, no chatbot can help | Same |
| MIV5.1 | -2 | No not really - it did make me want to smoke though | Smoke |
| MIV5.1 | -1 | Not particularly. Because it just repeated my answers back to me. | Same |
| MIV5.1 | -1 | No, the chatbot repeated information I gave back to me. All the things I wrote were already conclusions I had reached on my own. | Same |
| MIV5.1 | -1 | no, i already know i should make Quits but not ready now | Smoke |
| MIV5.1 | -1 | I think i should find another device to relax instead of smoking | Same |
| MIV5.1 | -1 | The conversation was pleasant, however, the chatbot repeated my theses. It was a positive experience, but it did not add much to the perception of my smoking. | Same |
| MIV5.1 | -1 | helped me realize how quickly I reach for a cigarette after waking up | Same |
| MIV5.1 | -1 | Yes :) it helped me think about the steps I would have to take if I wanted to quit smoking | Same |
| MIV5.1 | -1 | yes, it made me realize that if i stop smoking ont he pc it will stop rather quick | Same |
| MIV5.1 | -1 | I already knew why I smoke and also why should I stop. Didnt realize anything new. | Same |
| MIV5.1 | -1 | no, i just repeated the things i already knew | Same |
| MIV5.1 | -1 | Yes. It made me realize that I need more control over my smoking habits. | Same |
| MIV5.1 | -1 | It only made me remember stronger my vice that I have. | Smoke |
| MIV5.1 | -1 | maybe would make me think of looking into the costs. but i wouldnt say it made me commit to stop smoking or cut down | Same |
| MIV5.1 | -1 | No because I have discussed and thought about my smoking for decades. The chatbox didn't enlighten me further. | Same |
| MIV5.1 | 0 | Yes ... it confirmed that it's a habit I need to break. | Same |
| MIV5.1 | 0 | not really. it was quite short | Same |
| MIV5.1 | 0 | I was already aware of all of the things I stated however it was helpful to write it down | Same |
| MIV5.1 | 0 | Nothing I didn't already know I must say. I know smoking is a very bad habit and the faster you quit, the better. Still, it didn't make me realize anything in particular, but it was a reminder that I should quit smoking as soon as possible. | Same |
| MIV5.1 | 0 | not really | Same |
| MIV5.1 | 0 | Yes. I have put into words what I think almost every day. | Same |
| MIV5.1 | 0 | No, because I already knew all those things | Same |
| MIV5.1 | 0 | No,, i already knew everything i said | Same |
| MIV5.1 | 0 | It just reminded me of things I had already considered | Same |
| MIV5.1 | 0 | No, but I understand it's purpose in trying to help people stop smoking. | Same |
| MIV5.1 | 0 | yes it did i have to be the one to do it | Same |
| MIV5.1 | 0 | yes, gave me an insight of the dangers and made me realize that i am capable of quitting this behavior | Same |
| MIV5.1 | 0 | No, the bot wasn't very profound or intuitive. | Same |
| MIV5.1 | 0 | yes - that is my copying mechanism | Same |
| MIV5.1 | 0 | I have thought about quiting lots of times so i probably did not lealize anything new. | Same |
| MIV5.1 | 0 | no | Same |
| MIV5.1 | 0 | Noy really. Noy anything I already know. | Same |
| MIV5.1 | 0 | A little, seeing words written made me realize that i am a slave of myself | Same |
| MIV5.1 | 0 | No, as the bot only confirmed what I've already written down, so no breakthrough. | Same |
| MIV5.1 | 0 | Yes, it put it in another words so I could see it differently | Same |
| MIV5.1 | 0 | No. It wasn't able to understand my point of view and it didn't add anything, nor did it provide me with any new information. | Same |
| MIV5.1 | 0 | Made me realise the government will never make it legal | Same |
| MIV5.1 | 0 | YES I REALIZED THAT I DON'T WANT TO QUIT SMOKING. AS BAD AS IT IS | Same |
| MIV5.1 | 0 | Yes, it made me realize that I smoke for stress, to relax. | Same |
| MIV5.1 | 0 | Yes smoking is linked to stress | Same |
| MIV5.1 | 0 | Maybe, I just tried quit smoking a little while and I couldnt | Same |
| MIV5.1 | 0 | No, I have a feeling that nothing Quitd. | Same |
| MIV5.1 | 0 | Yes, it helped me to realize that I have a couple of priorities in changing my smoking behaviour. | Same |
| MIV5.1 | 1 | Honestly? It was just nice to talk to someone who understood what I was saying for once. But I didn't make any realizations. The bot is amazing though. | Same |
| MIV5.1 | 1 | Momentarily, yes. I ask myself similar questions from time to time, but lack the strong will and motivation to quit smoking cigarettes | Quit |
| MIV5.1 | 1 | It didn't because I don't believe a chatbot could motivate me | Same |
| MIV5.1 | 1 | no because it just reiterated what I had said previously | Same |
| MIV5.1 | 1 | It made me realize that I shouldn't and that i can't because it is an addiction with certain habits | Quit |
| MIV5.1 | 1 | It didn't, It just asked me questions I already knew the answer to and proceeded to say the same things with other words | Same |
| MIV5.1 | 1 | conversation did not help, because the bot only "understood" my problems but did not help in any way to solve them | Same |
| MIV5.1 | 1 | so helped me to see more clearly how this phenomenon negatively affects my life | Quit |
| MIV5.1 | 1 | Yes, it helped me see the bad consequences and effects of it | Quit |
| MIV5.1 | 1 | Wasn't very detailed, so didn't really make me think about quitting | Same |
| MIV5.1 | 1 | It didn't, I just answered questions I was asked | Same |
| MIV5.1 | 1 | no. it was, for me, just a questionaire | Same |
| MIV5.1 | 1 | quite honestly not, as I was only asked about my smoking habits. Since I work in the health area, I know perfectly well the harm that tobacco does to me and to make any Quit in this habit it has to come from myself because I have always known that it is not good for me. | Same |
| MIV5.1 | 1 | how much of my "peer pressure" was from me and not from my friends themselves | Quit |
| MIV5.1 | 1 | Nope, nothing was highlighted really that I didn't already know. I was mostly informing the chatbot | Same |
| MIV5.1 | 2 | Not really, I thought about those questions before | Same |
| MIV5.1 | 2 | not really, it did not include anything that I did not already know or give me ideas about quitting smoking | Same |
| MIV5.1 | 2 | yes because the chatbot said logical things for my health | Quit |
| MIV5.1 | 2 | Not really, im aware of How my whole situation looks and for now its the best option for me | Same |
| MIV5.1 | 2 | Yes it thought me that I need to reduce smoking. | Quit |
| MIV5.1 | 2 | No, nothing at all. The chatbot only really responded in order to clarify, it suggested nothing to help with quitting. | Same |
| MIV5.1 | 2 | Yes, it helped me realize that I often reach for a smoke when I get filled with anxiety. I really only smoke to help me cope with daily anxiety and restlessness. It gives me something to do with my hands which makes me feel like I am accomplishing something. | Quit |
| MIV5.1 | 3 | It kinda did but it was mostly just confirming the things I said | Same |
| MIV5.1 | 3 | Yes. Self analysis has made me understand. | Quit |
| MIV5.1 | 3 | A little bit. I know more what to do. | Quit |
| MIV5.1 | 3 | no because i alrwady knew all these | Same |
| MIV5.1 | 3 | yes, i am more concious about my health | Quit |
| MIV5.1 | 3 | no, the bot just rephrase my thinking | Same |
| MIV5.1 | 3 | not so much, they were things I knew and realized before | Same |
| MIV5.1 | 3 | Nothing I haven't already put together myself, I've already done a lot of self reflection over this so it has nothing to do with the bot. | Same |
| MIV5.1 | 3 | its sunk in more how addicted i am | Quit |
| MIV5.1 | 3 | it didn't , because the chatbox tried to understand my behavior, but didn't comment on it | Same |
| MIV5.1 | 3 | Not really, there were things that i have already known, so i hadn't learnt anything new about my smoking behavior | Same |
| MIV5.1 | 4 | It made me realise what I like and don't like about smoking and that I can do small things to reduce smoking | Quit |
| MIV5.1 | 4 | Not really, there was not any advice | Same |
| MIV5.1 | 4 | yes, that I deep down do not want to stop smoking yet. | Same |
| MIV5.1 | 4 | Not really, because this is a conversation that I have with myself daily | Same |
| MIV5.1 | 4 | Yes, he has a remorse that I smoke | Same |
| MIV5.1 | 4 | Yes of course it helps, he gave me advice to get what I want. | Quit |
| MIV5.1 | 4 | Yes, that I would feel better if I stopped smoking | Quit |
| MIV5.1 | 5 | One on hand , it did help me to listen to my thoughts and realise them. On the other hand, other than that, it did not provide any actual and useful solutions to my smoking problem. | Same |
| MIV5.1 | 5 | It was helpful just to talk and realize how I feel about my own smoking. | Same |
| MIV5.1 | 5 | I need time to endure I follow through | Same |
| MIV5.1 | 5 | Kind of. It put what I want and need into perspective. | Quit |
| MIV5.1 | 6 | It made me think more seriously about my desire to quit | Quit |
| MIV5.1 | 6 | It made me think I have to stop smoking. Look for ways to do so. | Quit |
| MIV5.1 | 6 | The conversation didnt Quit how I felt however thinking about smoking more and thinking about quitting makes me feel more guilty about the fact i havent made an effort to reduce my smoking habits | Quit |
| MIV5.1 | 7 | Not alot like I said I was just starting to think about it | Same |
| MIV5.1 | 7 | Yes. That I can set a goal on how many I can smoke per day. | Quit |
| MIV5.1 | 7 | Partly, because i already know what to do, it is just hard to stop smoking. | Same |
| MIV5.2 | -6 | That i might not have the willpower and the mentality to cut it off so easily | Smoke |
| MIV5.2 | -4 | Yes that I want to make a Quit, that I am not ready to quit and that it is sad that one of the few things in my life at the moment that make me feel comfort is smoking. | Smoke |
| MIV5.2 | -3 | It just helped me to think about quitting smoking again, but although I want to, at this point in my life it is very difficult. | Smoke |
| MIV5.2 | -2 | Yes. That I smoke even when I don't need it. | Same |
| MIV5.2 | -2 | nope, it was just simple smalltalk on what could be done for me to stop smoking | Same |
| MIV5.2 | -1 | Not really, the conversation merely confirmed what I knew already, it did not bring up anything new | Same |
| MIV5.2 | -1 | It seemed to repeat things i already knew but in a medical context and i'm hyper aware already so its hard to know what to do next, it tried to help but it didnt tell me things i wasn't already thinking about, my anxiety and pain are severe and without guidance im lost | Smoke |
| MIV5.2 | -1 | it didn't make me realise anything new, the chat bot simply repeated my points and gave some basic input. | Same |
| MIV5.2 | -1 | no, im fully aware of my addiction and behaviors connected with it | Smoke |
| MIV5.2 | -1 | Nothing at all. | Same |
| MIV5.2 | -1 | yes, because it made me rethink some of my behaviors torwards smoking | Same |
| MIV5.2 | -1 | ye a bit, bot countinously asked me questions that made me think about that for a couple of seconds and make good arguments based on my responses | Same |
| MIV5.2 | -1 | i already thought about that but telling it made me think more | Same |
| MIV5.2 | -1 | I think yes. I found out that probably i smoke more than regular smoker | Same |
| MIV5.2 | -1 | No, because even though I would like to smoke less, I don't want to quit because it helps me | Smoke |
| MIV5.2 | -1 | Not really, because those were all conclusions I had already reached by myself | Same |
| MIV5.2 | 0 | It helped me realize I need to start to try and understand myself better so I can quit bad habits such as smoking | Same |
| MIV5.2 | 0 | that I smoke a little bit more than I thought | Same |
| MIV5.2 | 0 | It did not, I know my smoking is a terrible habit and I know that I need to stop, I have tried multiple times with a variety of methods without luck. There was no further information given to me through the chat bot that I don't already know. | Same |
| MIV5.2 | 0 | No, It only helped to confirm what I already knew about my smoking, but did not give me any new insight | Same |
| MIV5.2 | 0 | A little, it made me think that I smoke too much | Same |
| MIV5.2 | 0 | Yes, that the central problem is my anxiety and that I should prioritize that. | Same |
| MIV5.2 | 0 | I'm pretty self aware of my smoking habits, so I don't think I gained any new insight into it. I already know quite well what I should do and even how to achieve my goal of reducing smoking, but I feel like I lack the motivation to do so. I'm not sure if the chatbot actually suggested anything, it only asked questions, which to be fair, can be quite useful for others. | Same |
| MIV5.2 | 0 | It did so in a relevant way. | Same |
| MIV5.2 | 0 | Nothing I am not already thinking. | Same |
| MIV5.2 | 0 | It helped me realize that smoking offers me nothing at all really | Same |
| MIV5.2 | 0 | not really, there are things that already know | Same |
| MIV5.2 | 0 | I don't think so. I know I wanna quit smoking, the conversation didn't help me to realise that | Same |
| MIV5.2 | 0 | Yes, it made me realize the emphasis stress plays in my smoking habits. | Same |
| MIV5.2 | 0 | I think so. It made me think about my smoking | Same |
| MIV5.2 | 0 | Yes. It helped me realize I need a strategic plan to stop smoking for good. | Same |
| MIV5.2 | 0 | No, im very self aware of my habbit, im aware of the health problems it may give me and the waste of money it is | Same |
| MIV5.2 | 0 | not really | Same |
| MIV5.2 | 0 | Yes, it made me realize that i don't know which steps to follow in order to help with quitting my smoking | Same |
| MIV5.2 | 0 | yes, that I actually go outside to get some fresh air and a Quit of scenery | Same |
| MIV5.2 | 0 | yes, i should smoke less | Same |
| MIV5.2 | 0 | It helped me to realize that smoking is having an effect on my health and on my finances because I had to take the time to think of ways that smoking is affecting my life and things I can do to quit smoking | Same |
| MIV5.2 | 0 | yes made me realize some things about my smoking habit | Same |
| MIV5.2 | 0 | I'm generally very aware about my smoking habit, but it was interesting conversing about it | Same |
| MIV5.2 | 1 | Not so much | Same |
| MIV5.2 | 1 | More or less. I already know that I should stop smoking, but the conversation didn't gave me any reasons or ideias to stop | Same |
| MIV5.2 | 1 | No, as I'm already aware of the cons of smoking. | Same |
| MIV5.2 | 1 | Yes it was helpful to rationalise why I smoke and what should I do to start smoking less | Quit |
| MIV5.2 | 1 | Not really, I am mindfull of my addiction | Same |
| MIV5.2 | 1 | Not much; there were questions inquiring about habits I have already acknowledged | Same |
| MIV5.2 | 1 | Yes it did, I had to think about the reasons of my addiction | Quit |
| MIV5.2 | 1 | The conversation makes me realize that i am very addicted. | Quit |
| MIV5.2 | 1 | That just reminds me why I want to quit. | Same |
| MIV5.2 | 1 | it did not help | Same |
| MIV5.2 | 1 | Yes, it made me realize I need to Quit | Quit |
| MIV5.2 | 1 | no, I already knew | Same |
| MIV5.2 | 1 | no i did not realize anything new | Same |
| MIV5.2 | 1 | yes, it made me give deeper thought into my habits and the effects it has in other parts of my life | Quit |
| MIV5.2 | 1 | i realized im surrounded by the wrong crowd | Quit |
| MIV5.2 | 1 | Not really. | Same |
| MIV5.2 | 1 | No, I have been a regular smoker for 64 years and know all about it | Same |
| MIV5.2 | 1 | I think it could give me some courage to do something and finally manage to stop smoking. But it didn't really give me any information that I didn't knew already. | Quit |
| MIV5.2 | 1 | Not really , I knew I need to set goals | Same |
| MIV5.2 | 1 | No. I am aware I should quit. I am aware of what I like and what I don't like. Moreover, my addiction is not about nicotine, purely about the act - I didn't light a single cig for a week now because it's cold and smoking would be unpleasant. | Same |
| MIV5.2 | 1 | no, because I already knew this behavior | Same |
| MIV5.2 | 2 | Yes, it helped me understand why do I smoke | Quit |
| MIV5.2 | 2 | Not really, as I stated before i felt like the chat bot just repeated whatever i told it adding no extra input, in a way it just felt like I was talking in the mirror. | Same |
| MIV5.2 | 2 | Yes, if I do things gradually it is probably achievable. | Quit |
| MIV5.2 | 2 | Yes, I have realized that I smoke too much and if I don't quit I might die of lung cancer or disease. | Quit |
| MIV5.2 | 2 | No, because I am already aware of my smoking habit | Same |
| MIV5.2 | 2 | It didn't help me realize anything because i was aware of consequences of my habit, but it definetly make me think about quitting smoking | Quit |
| MIV5.2 | 2 | No. I had already realised that I have a smoking addiction | Same |
| MIV5.2 | 2 | He just copied most of the things i wrote, so I felt like I was talking to myself | Same |
| MIV5.2 | 2 | Yes, it made me realise things I was not really taking into consideration when I am smoking. | Quit |
| MIV5.2 | 2 | Perhaps, the conversation begins to make me think about this vice. | Same |
| MIV5.2 | 2 | Yes, that quitting smoking is a life-long process and it will need to be a gradual Quit. | Quit |
| MIV5.2 | 2 | Not really, it's something I have been monitoring for a while now | Same |
| MIV5.2 | 3 | Yes it made me realize that smoking is a bad habit through the questions that I was asked | Quit |
| MIV5.2 | 3 | Yes. It helped incorporate the ideal that the Quit I'll have in my life from quitting smoke is for the best. | Quit |
| MIV5.2 | 3 | Not really, I am already aware of my bad habits and the health factors associated with it, nothing really new was stated | Same |
| MIV5.2 | 3 | Yes it did, I have realized that now I smoke more than I did a month ago. | Quit |
| MIV5.2 | 3 | Yes, it made me realise I do it for the constant need of pleasure, which hadn't occurred to me | Quit |
| MIV5.2 | 3 | Yes a bit, i noticed i don't have many things that i like about smoking, it's only addiction. | Quit |
| MIV5.2 | 3 | maybe to reflect about that and aspire to improve and to a better situation | Quit |
| MIV5.2 | 3 | Kinda, now i still think about my girlfriend that i want her to quit smoking too. It's make it kinda easier mentally. | Quit |
| MIV5.2 | 3 | It helped me realise that as soon as i quit the habit of social smoking , i will be one step closer to quit smoking once and for all. | Quit |
| MIV5.2 | 3 | No. It didnt gave me any advice how to stop smoking | Same |
| MIV5.2 | 3 | I was completely aware of my behavior already | Same |
| MIV5.2 | 3 | Yes | Same |
| MIV5.2 | 3 | no, because it suggested nothing it was just my own answers | Same |
| MIV5.2 | 3 | It was nice to type out my thoughts and be asked about my habits. | Quit |
| MIV5.2 | 3 | Yeah, I have the courage to quit it, but after some time I get back by the feeling of calm that the nicotine prodeces on me. | Same |
| MIV5.2 | 4 | yes it made me realise that i can quit and that im not really keen on smoking anyway im just addicted | Same |
| MIV5.2 | 4 | it elaborated what I meant in a kind way | Same |
| MIV5.2 | 4 | Yes. It did help me realizing how bad I should leave this addiction behind. | Quit |
| MIV5.2 | 4 | No, not really the conversation was not that deep, but afterwards i was more determined to stop and to give it a try. | Quit |
| MIV5.2 | 5 | Well since I am trying to quit, I already make sense of my smoking behaviors. | Same |
| MIV5.2 | 5 | Yeah, mine is very addictive and it needs to stop | Quit |
| MIV5.2 | 5 | Yes because now i have realized that it is easy stop smoking if you willing to | Quit |
| MIV5.2 | 5 | Yes. I only realized now that smoking is a expensive habit. | Quit |
| MIV5.2 | 6 | Yes. The questions made me think about smoking as an addiction and that's something I don't usually consider | Quit |
| MIV5.2 | 6 | It was interesting to see it in writing but the chat bot (as lovely as they were) simply rephrased/clarified what I had just told them so I didn't learn anything new. | Same |
| MIV5.2 | 6 | No, it just aske me questions | Same |
| MIV5.2 | 8 | yes, It made me realise my smoking habits are not good and need to be Quitd. | Quit |
| MIV5.2 | 10 | Yes it did because it helped me do some self introspection . | Quit |
